# Noncanonical role of KDM5C in conferring bortezomib resistance via the PERK‒Nrf2 axis in multiple myeloma

**DOI:** 10.1038/s41419-026-08591-7

**Published:** 2026-03-23

**Authors:** Peifen Lu, Wenbin Shangguan, Weiwei Qian, Dongliang Wu, Wenyang Li, Jingjing Huang, Peipei Xu, Dijun Chen, Feng Li, Bing Chen, Quan Zhao

**Affiliations:** 1 The State Key Laboratory of Pharmaceutical Biotechnology, Department of Hematology, the Afﬁliated Drum Tower Hospital of Nanjing University Medical School, China–Australia Institute of Translational Medicine, School of Life Sciences, Nanjing University, Nanjing, China; 2 School of Clinical and Basic Medical Sciences, Shandong First Medical University & Shandong Academy of Medical Sciences, Jinan, China; 3 Department of Geriatrics, The Fourth Affiliated Hospital of Nanjing Medical University, Nanjing, China; 4 Department of Hematology, Jinling Hospital, School of Medicine, Nanjing University, Nanjing, China

**Keywords:** Oncogenes, Mechanisms of disease

## Abstract

Conventionally, KDM5C functions as a specific demethylase that targets histone H3 lysine 4 dimethyl and trimethyl modifications, crucial for gene expression. However, the role of KDM5C in multiple myeloma (MM) progression and bortezomib (BTZ) resistance has remained elusive. In this study, we found noncanonical functions of KDM5C in MM. Specifically, KDM5C binds to CBP and MYC, conferring BTZ resistance in MM through a demethylase-independent mechanism. Our investigations revealed that KDM5C is markedly upregulated in BTZ-resistant MM patients as well as those with relapsed MM. Significantly, the expression level of KDM5C exhibits an inverse correlation with the overall survival of MM patients. Moreover, KDM5C is indispensable for MM cell proliferation. Depletion of KDM5C augmented the sensitivity of MM cells to BTZ treatment both in vitro and in vivo. We found that KDM5C forms a novel complex with CBP and MYC via its PHD2 domain. This complex formation triggers lysine 27 acetylation in histone H3 (H3K27ac) and subsequent enrichment of H3K27ac on the PERK promoter. As a result, PERK transcription is activated, and Nrf2 phosphorylation is promoted, bolstering the unfolded protein response within the endoplasmic reticulum of MM cells. In contrast, the methylation status of histone H3 lysine 4 (H3K4me1/3) on the PERK promoter remains unaltered, regardless of the complex state. Taken together, the findings of this study underscore the key role of KDM5C as a driving force behind MM progression and BTZ resistance, indicating that KDM5C represents a novel and promising therapeutic target for the treatment of BTZ-resistant MM.

## Introduction

Multiple myeloma (MM), the second most prevalent hematological malignancy, accounts for approximately 15% of all hematological malignancies worldwide [1]. Over the past decade, the application of proteasome inhibitors like bortezomib (BTZ) has significantly improved the overall survival rate of myeloma patients. However, acquired resistance to BTZ is an inevitable clinical outcome, leading to treatment failure in relapsed and refractory cases [2]. Thus, elucidating its pathogenesis and resistance mechanisms is crucial for developing more effective therapies.

Tumorigenesis and progression in MM are driven by genetic and epigenetic alterations. Clinically, ~40% of MM patients have IgH translocations at 14q32, which accelerates tumor progression and confer resistance [3]. A key genetic event in MM development is c-MYC overexpression, driven by myc locus translocation/amplification or upstream pathway activation [3]. MYC serves as a pivotal transcription factor, and its high expression drives cancer progression by amplifying target gene transcription.

Epigenetic modifications, such as DNA methylation and histone alterations, significantly impact MM progression. One of the most well-defined epigenetic marks is trimethylated lysine 4 on histone H3 (H3K4me3), whose dysregulation is frequently associated with MM tumor progression and drug resistance [4]. KDM5C, also known as JARID1C or SMCX, is responsible for demethylating H3K4me2/3 and belongs to the KDM5 demethylase subfamily. Other members of the KDM5 family, including KDM5A (JARID1A/RBP2), KDM5B (JARID1B/PLU-1), and KDM5D (JARID1D/SMCY), also participate in the demethylation of H3K4me2/3 [5]. Among them, KDM5D has been identified as a Y chromosome gene [6]. Mounting evidence suggests that KDM5C represents a potential target for molecular-based cancer therapies. In clear cell renal cell carcinoma, KDM5C acts as a tumor suppressor, and its inactivation leads to heterochromatin noncoding RNA activation and increased cancer cell invasiveness [7]. The role of KDM5C in breast cancer remains unclear. It has been reported as both an oncogene (enhancing invasion and inhibiting STING expression) [8–10] and a tumor suppressor (preventing oncogene enhancer overactivation) [11]. Nevertheless, KDM5C plays an oncogenic role in gastric cancer [12], colorectal cancer [13–15], prostate cancer [16–18] and hepatocellular carcinoma [19, 20]. In addition to its role in cancer, KDM5C is implicated in X-linked mental retardation (XLMR), with mutations driving XLMR through both enzyme activity inactivation and non-enzyme activity region mutations [21, 22], implying its biological function may not rely solely on its enzyme activity.

In the present study, we identified KDM5C as a crucial oncogenic factor in MM progression and BTZ resistance. We demonstrated that elevated expression of KDM5C was specifically correlated with MM progression, bortezomib resistance, and poor prognosis in MM patients. Depletion of KDM5C impeded MM tumor growth and sensitized MM cells to BTZ treatment both in vitro and in vivo. Surprisingly, KDM5C directly activated the PERK‒Nrf2 axis to augment endoplasmic reticulum (ER) stress in a demethylase-independent manner. We discovered that KDM5C forms a novel complex with MYC and CBP and that this complex is essential for regulating H3K27ac levels and enriching the PERK promoter to modulate ER stress in MM. Thereby revealed a key oncogenic driver role of KDM5C in MM progression and BTZ resistance.

## Results

### High expression of KDM5C is correlated with bortezomib resistance and shorter survival in MM patients

**Fig. 1:**
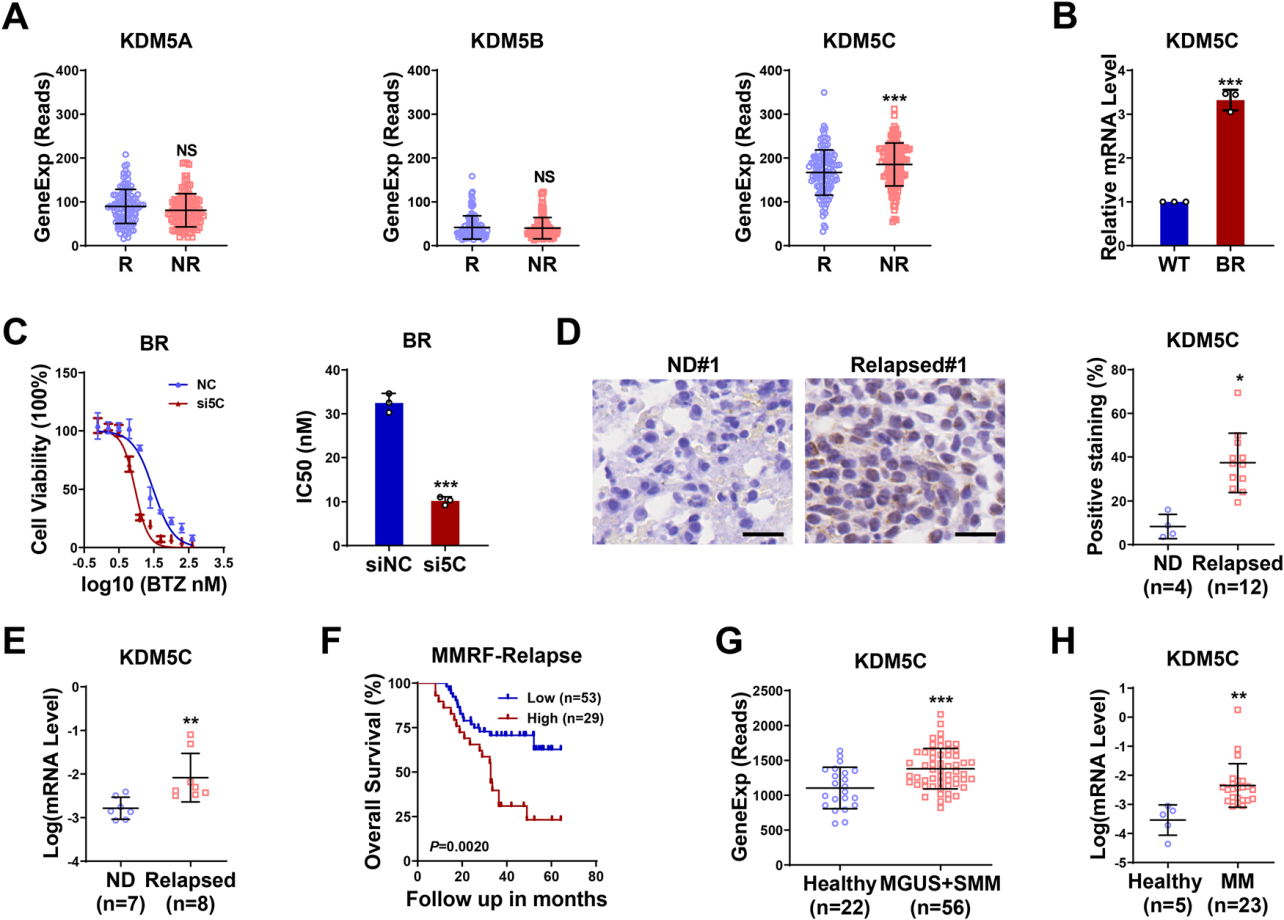
Highly expressed KDM5C is associated with BTZ resistance and poor outcomes. **A** KDM5A, KDM5B and KDM5C expression in patients who responded (R, *n* = 84) or did not respond (NR, *n* = 125) to BTZ-based regimens in the GSE2658 cohort. **B** KDM5C expression in BTZ-resistant cells (BR) compared with parental LP-1 cells (WT). **C** The IC_50_ of BTZ in BTZ-resistant (BR) cells after silencing KDM5C expression (si5C). **D** Representative IHC results of KDM5C staining and statistical data for newly diagnosed (ND) and relapsed MM patient bone marrow (relapsed). Scale bar = 20 μm. **E** KDM5C expression in CD138^+^ cells collected from 7 newly diagnosed (ND) and 8 relapsed MM patients. **F** Kaplan–Meier survival analyses for KDM5C expression in MM-relapsed patients in the MMRF CoMMpass datasets. **G** KDM5C expression in a cohort of healthy donor controls (*n* = 22) and monoclonal gammopathy of undetermined significance (MGUS) and smoldering multiple myeloma (SMM) patient samples (*n* = 56) from the GES5900 dataset. **H** KDM5C expression in CD138^+^ bone marrow cells collected from 5 healthy donor controls and 23 MM patients. *P* values were determined by Student’s t test (A-E, G and H) or Pearson’s coefficient and log-rank test (F). *P < 0.05; **P < 0.01; ***P < 0.001; NS not significant.

Accumulating evidence indicates an essential role of the KDM5 family of demethylases in cancer [5, 23]. However, the biological impact of KDM5 family members on BTZ resistance in MM treatment remains unknown. To investigate the BTZ-responsive clinical relevance of KDM5C in MM, we first analyzed public datasets of MM patients with BTZ responsiveness. GSE9782 dataset analysis revealed that the expression levels of KDM5C in patients who responded to BTZ-based regimens (R) were significantly lower than those in patients who did not respond to BTZ (NR), whereas the expression levels of KDM5A and KDM5B were similar between the R and NR groups (Fig. 1A). Accordingly, analysis of data from relapsed patients in the coMMpass database revealed that KDM5C was significantly more highly expressed in MM patients than KDM5A or KDM5B (Supplementary Fig. S1A), suggesting that KDM5C is crucial for MM progression and BTZ responsiveness.

Next, we examined the expression levels of KDM5C in an established bortezomib-resistant MM cell line (BR) derived from parental LP-1 cells (WT) (Supplementary Fig. S1B). As shown in Fig. 1B, KDM5C was indeed expressed at significantly higher levels in BR cells than in WT. We subsequently tested the role of KDM5C in BTZ resistance. When KDM5C was depleted, BTZ sensitivity in BR cells significantly increased (Fig. 1C and Supplementary Fig. S1C), indicating that KDM5C may play a key role in BTZ resistance.

We further analyzed KDM5C levels in newly diagnosed and relapsed patients in our cohort of MM patients. We found that KDM5C staining was significantly greater in the bone marrow of relapsed patients than in that of newly diagnosed patients (Fig. 1D and Supplementary Fig. S1D). Consistently, KDM5C mRNA levels in relapsed patient–derived CD138^+^ cells were significantly greater than those in newly diagnosed patients (Fig. 1E). In addition, we found that relapsed patients with higher KDM5C expression had significantly shorter overall survival than did those with lower KDM5C expression (Fig. 1F). In agreement with these results, we found that KDM5C expression levels were significantly higher in patients with monoclonal gammopathy of undetermined significance (MGUS) and smoldering multiple myeloma (SMM) than in healthy donors in the GSE5900 dataset (Fig. 1G). Furthermore, we collected bone marrow from 5 healthy donors and 23 MM patients and showed that KDM5C expression levels in CD138^+^ cells were significantly greater than those in healthy control bone marrow cells (Fig. 1H). More importantly, higher levels of KDM5C correlated with shorter overall survival in patients with MM (Supplementary Fig. S1E). Together, these results suggest a role for KDM5C in promoting the malignant progression of MM and BTZ resistance.

### KDM5C is required for MM cell proliferation and progression

**Fig. 2:**
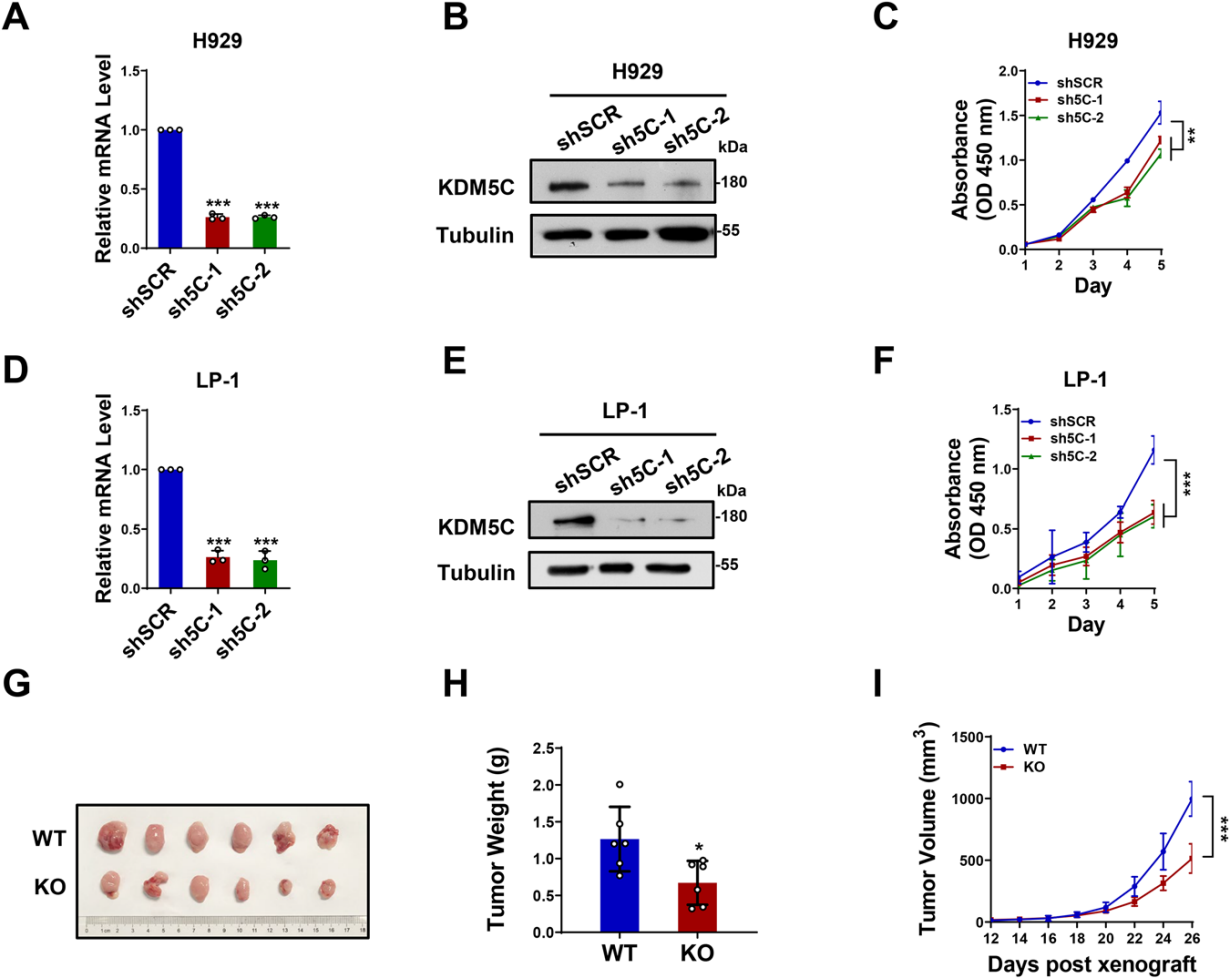
KDM5C is required for MM cell proliferation and tumor growth. **A,** **B** The mRNA and protein levels of KDM5C after transduction with the indicated shRNAs (sh5C-1, sh5C-2) relative to shSCR in H929 cells. **C** Growth curve of H929 cells with KDM5C knockdown (sh5C-1, sh5C-2). **D**, **E** The mRNA and protein levels of KDM5C after transduction with the indicated shRNAs (sh5C-1, sh5C-2) relative to shSCR in LP-1 cells. **F** Growth curve of LP-1 cells with KDM5C knockdown (sh5C-1, sh5C-2). Photographs (**G**) and weights (**H**) of tumors at 26 days after subcutaneous injection. **I** Tumor growth curves of WT and KO cell-derived xenografts. *P* values were determined by Student’s *t* test. *P < 0.05; **P < 0.01; ***P < 0.001.

Next, we sought to determine whether KDM5C contributes to MM cell proliferation and progression. We first knocked down KDM5C expression in H929 cell lines (Fig. 2A, B). We found that KDM5C depletion significantly inhibited cell growth (Fig. 2C). Notably, knocking down KDM5C did not affect cell apoptosis (Supplementary Fig. S2A). Similarly, knocking down KDM5C in another MM cell line, LP-1, produced similar results (Fig. 2D–F and Supplementary Fig. S2B). Moreover, we established a KDM5C knockout (KO) cell line in which KDM5C had a similar effect on MM cell growth (Supplementary Fig. S2C–E).

To determine whether KDM5C is required for MM progression in vivo, H929 KDM5C-wild-type (WT) and KDM5C-knockout (KO) cells were injected into NSG mice subcutaneously, and the tumor volume was recorded. We found that tumor growth in the KDM5C-KO xenografts was significantly slower (Fig. 2G, H). Consistently, the tumor volume was significantly smaller in KDM5C-KO xenografts (Fig. 2I). Notably, the body weights remained stable during the experiment (Supplementary Fig. S2F). Collectively, our results indicate a role for KDM5C in promoting MM cell proliferation and progression.

### KDM5C targets the PERK-Nrf2 axis in MM

**Fig. 3:**
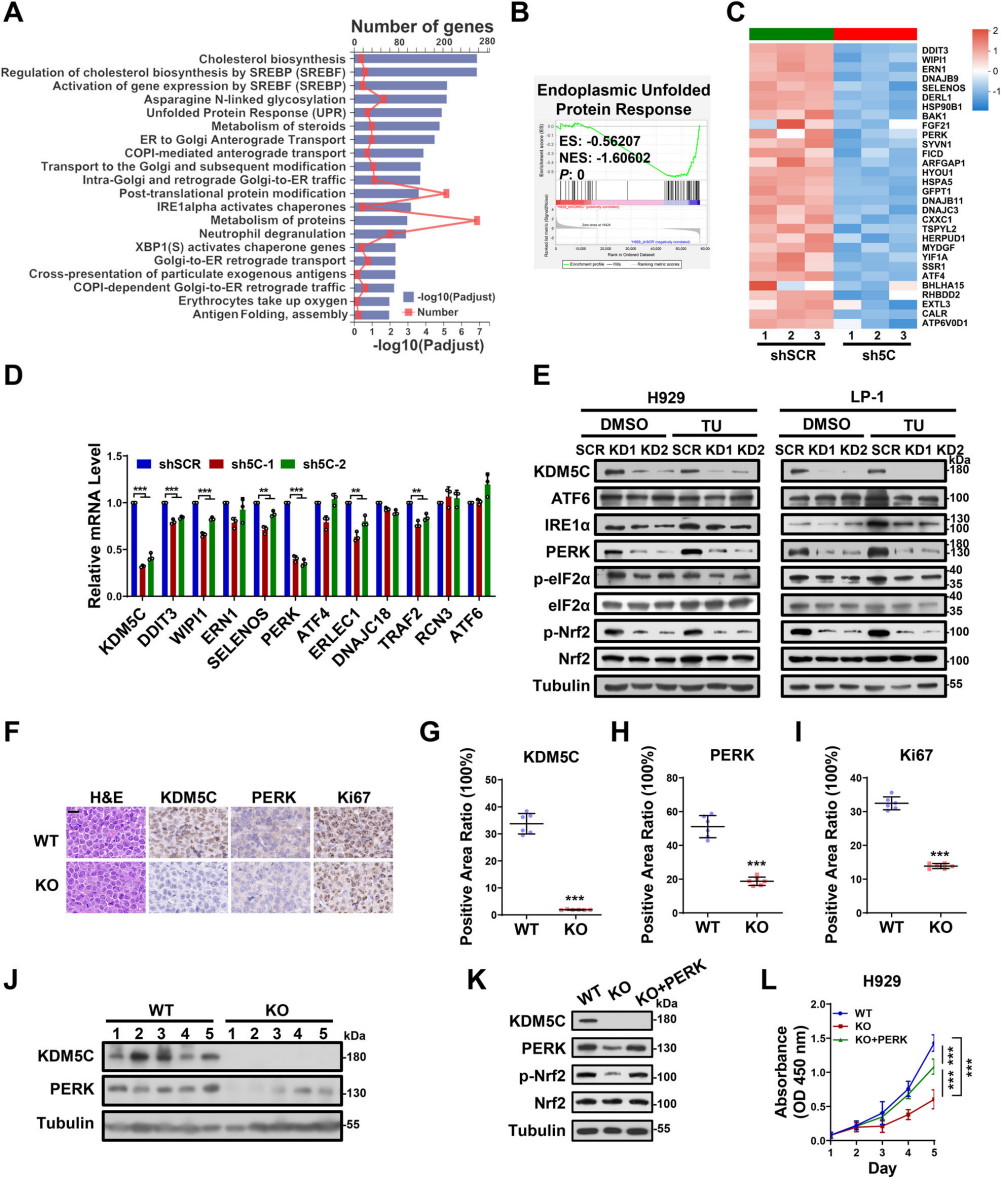
KDM5C targets the PERK‒Nrf2 axis in MM. **A** Reactome enrichment analysis of DEGs (differentially expressed genes) between the control (shSCR) and KDM5C-knockdown (sh5C) groups. **B** GSEA enrichment score curves. **C** Heatmap of the top 30 DEGs from Fig. 4B gene set. **D** mRNA levels of ER stress-related genes after knocking down KDM5C (sh5C-1, sh5C-2) relative to SCR in H929 cells. **E** The protein levels of several key proteins involved in ER stress in H929 and LP-1 cells in which KDM5C was knocked down (KD) relative to those in the SCR with or without tunicamycin treatment. TU: tunicamycin, 10 μg/mL for 6 h. **F** IHC of protein stains of KDM5C, PERK and Ki67 in the KDM5C-KO xenografts compared with the WT xenografts as in Fig. 2G. **G**– **I** Statistical graph of the IHC results in (**F**). **J** KDM5C and PERK expression in KDM5C-KO xenografts compared with WT xenografts. **K** The protein level of PERK-p-Nrf2 in KDM5C-KO H929 cells after PERK expression was restored (KO + PERK). **L** Growth rate of KDM5C-KO H929 cells after PERK expression was restored (KO + PERK). ES, enrichment score; NES, normalized enrichment score. *P* values were determined by Student’s *t* test. **P < 0.01; ***P < 0.001.

To explore the molecular mechanisms of KDM5C in MM progression, we performed RNA-seq to profile the transcriptomes of H929 cells after KDM5C knockdown. In total, 1120 differentially expressed genes (DEGs) were downregulated, whereas 1346 DEGs were upregulated upon KDM5C knockdown (|FC|>1.2, *P* < 0.01) (Supplementary Fig. S3A, B). Reactome enrichment revealed that these DEGs were enriched mostly in response to ER stress and downstream signaling pathways, including the unfolded protein response (UPR), cholesterol biosynthesis, protein transportation and metabolism (Fig. 3A). Notably, ER stress plays an important role in the development of a variety of cancers, especially MM, which is characterized by extensive immunoglobulin production, leading to an excessive load on protein homeostasis in tumor cells [24]. Gene set enrichment analysis (GSEA) further confirmed that KDM5C was highly associated with the expression of genes involved in the UPR of the ER (Fig. 3B and Supplementary Fig. S3C). Figure 3C shows the top 30 DEGs from the UPR/ER stress gene set shown in Fig. 3B. qRT‒PCR was subsequently performed to validate the effects of KDM5C on those specific selective genes. As shown in Fig. 3D, KDM5C-KD significantly reduced the expression levels of mRNAs encoding ER stress-related genes, such as DDIT3, WIPI1, SELENOS, PERK, ERLEC1, DNAJC18 and TRAF2, whereas those encoding ERN1, ATF4, RCN3 and ATF6 remained unchanged. Among these genes, PERK was the most downregulated gene upon KDM5C knockdown (Fig. 3D, Supplementary Fig. S3D). We further examined protein level changes of major players involved in the UPR/ER stress pathway (UPR^ER^) with or without tunicamycin (a well-characterized inducer of ER stress) treatment. Consistent with these findings, we detected a significant decrease in PERK protein expression in KDM5C-KD cells in the presence or absence of tunicamycin (Fig. 3E). These findings further confirm that KDM5C plays a critical role in modulating the ER stress response in MM cells by regulating the PERK–Nrf2 axis of the UPR/ER stress pathway. Interestingly, the phosphorylation levels of eIF2α and Nrf2, which are key downstream effectors of the PERK kinase [24, 25], were significantly reduced in KDM5C-knockdown cells. Consistent with this, the expression of genes downstream of phosphorylated Nrf2 (p-Nrf2), including HO-1 and NQO1, was also markedly decreased (Fig. 3E and Supplementary Fig. S3E). Many studies have demonstrated that Nrf2 activation contributes to proteasome inhibitor resistance and aberrant cell proliferation in MM [26–28]. Since the expression of ERN1 (encoding the Ire1 protein), ATF6 and ATF4 did not change upon KDM5C knockdown, these results indicate that KDM5C might mediate the UPR^ER^ through the PERK‒Nrf2 axis in MM cells.

To further test the effect of KDM5C on PERK expression during MM progression in vivo, we examined the expression of PERK in KDM5C-KO xenografts. As shown in Fig. 3F–J, the expression of PERK was significantly lower in the KDM5C-KO xenografts than in the WT xenografts. In addition, we exogenously enforced PERK expression in KDM5C-KO cells to perform rescue experiments for cell growth. As shown in Fig. 3K, after exogenous PERK expression, the phosphorylation of Nrf2 (p-Nrf2) was significantly restored, as was cell proliferation (Fig. 3L), indicating that PERK is the key downstream gene of KDM5C during MM progression. Together, these results suggest that KDM5C is required to maintain the UPR^ER^ in MM cells and that PERK is a key mediator that modulates the function of KDM5C in ER stress to promote MM progression.

### KDM5C contributes to bortezomib resistance in MM cells

**Fig. 4:**
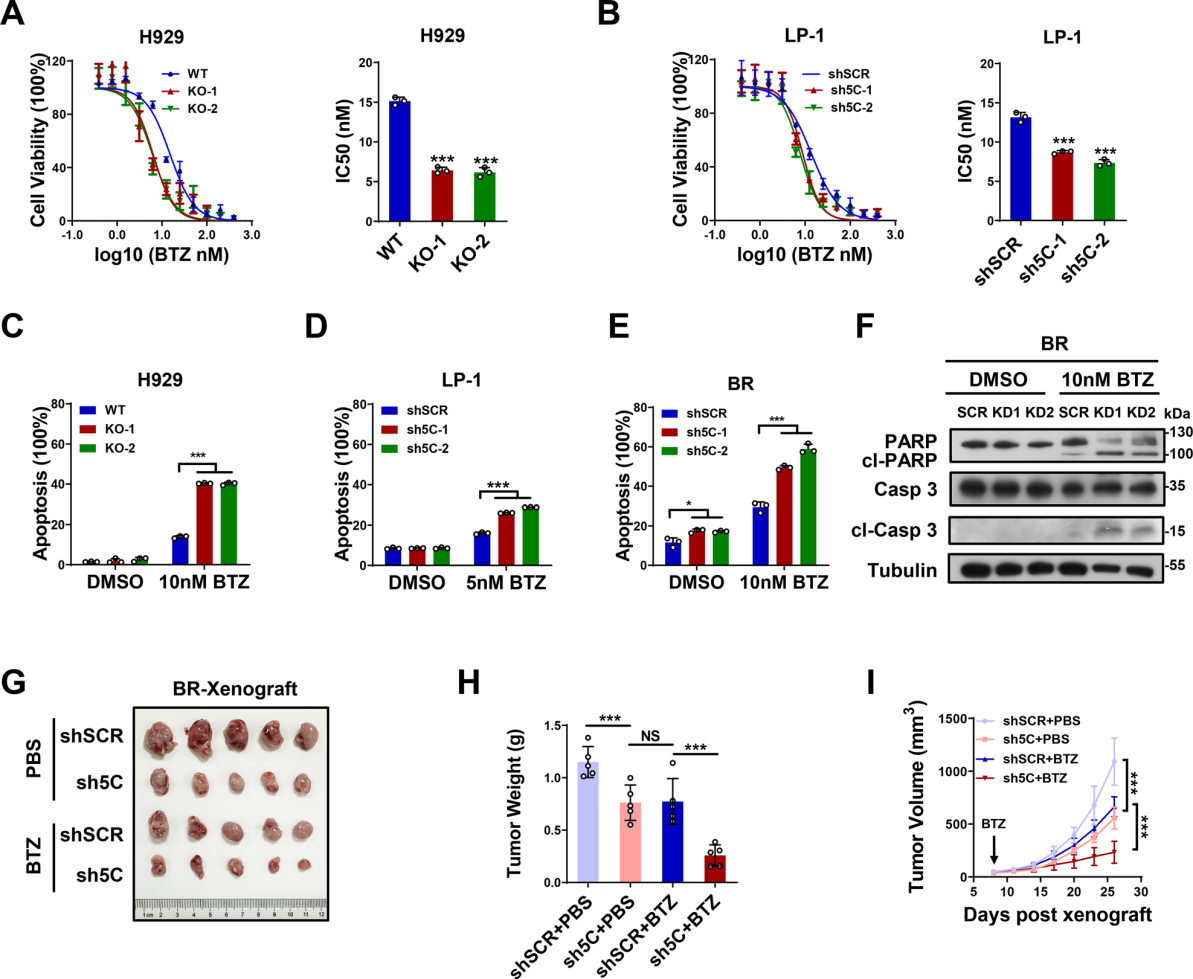
KDM5C contributes to bortezomib resistance in MM cells. **A** The IC_50_ of BTZ in H929 cells after knockout of KDM5C (5C-KO-1, 5C-KO-2). **B** The IC_50_ of BTZ in LP-1 cells after knocking down KDM5C (sh5C-1, sh5C-2). Apoptosis rates from FACS in KDM5C-knockout H929 (**C**) and LP-1 (**D**) cells with KDM5C knockdown (sh5C-1, sh5C-2) induced by BTZ. **E** Apoptosis rates determined via FACS in KDM5C-knockdown (sh5C-1, sh5C-2) BTZ-resistant (BR) cells induced with BTZ. **F** The levels of cleaved PARP and caspase 3 in BTZ-resistant (BR) cells with KDM5C knockdown (sh5C-1, sh5C-2) induced by BTZ. Photographs (**G**) and weights (**H**) of tumors from BR cell-derived xenografts with or without BTZ treatment. **I** Tumor growth curves. *P* values were determined by Student’s *t* test (A-E) and two-way ANOVA (**H**–**I**). ***P < 0.001; NS not significant.

Next, we aimed to determine whether KDM5C is required for BTZ resistance in MM. First, we explored whether KDM5C mediates the sensitivity of H929 and LP-1 MM cells to bortezomib. As shown in Fig. 4A, the IC_50_ of BTZ in H929 cells was significantly reduced upon KDM5C knockout (KO-1 and KO-2). Similar results were observed for the KDM5C-knockdown (Sh5C-1 and sh5C-2) LP-1 and H929 cells (Fig. 4B and Supplementary Fig. S4A). In addition, the increase in sensitivity to BTZ was confirmed by the significant increase in apoptosis rates induced by BTZ in KDM5C-KO H929 (Fig. 4C and Supplementary Fig. S4B), KDM5C-KD LP-1 (Fig. 4D and Supplementary Fig. S4C) and KDM5C-KD H929 (Supplementary Fig. S4D) cells. Consistently, increases in cleaved poly (ADP‒ribose) polymerase (PARP) and cleaved caspase 3 were observed in KDM5C-KD LP-1 (Supplementary Fig. S4E) and KDM5C-KD H929 cells (Supplementary Fig. S4F) upon BTZ treatment. Furthermore, we established stable KDM5C-knockdown BR cells (Supplementary Fig. S4G), and the rates of apoptosis induced by BTZ were significantly increased by BTZ treatment (Fig. 4E and Supplementary Fig. S4H), accompanied by increased levels of cleaved PARP and cleaved caspase 3 (Fig. 4F). Moreover, we evaluated the in vivo effects of KDM5C on resistance to BTZ treatment via BR cell-derived xenografts. We found that KMD5C depletion further inhibited MM tumor growth, suggesting that KMD5C depletion sensitized MM xenografts to BTZ treatment (Fig. 4G–I and Supplementary Fig. S4I). These results indicate that KDM5C contributes to resistance to bortezomib in MM cells.

### KDM5C confers bortezomib resistance via PERK in MM

**Fig. 5:**
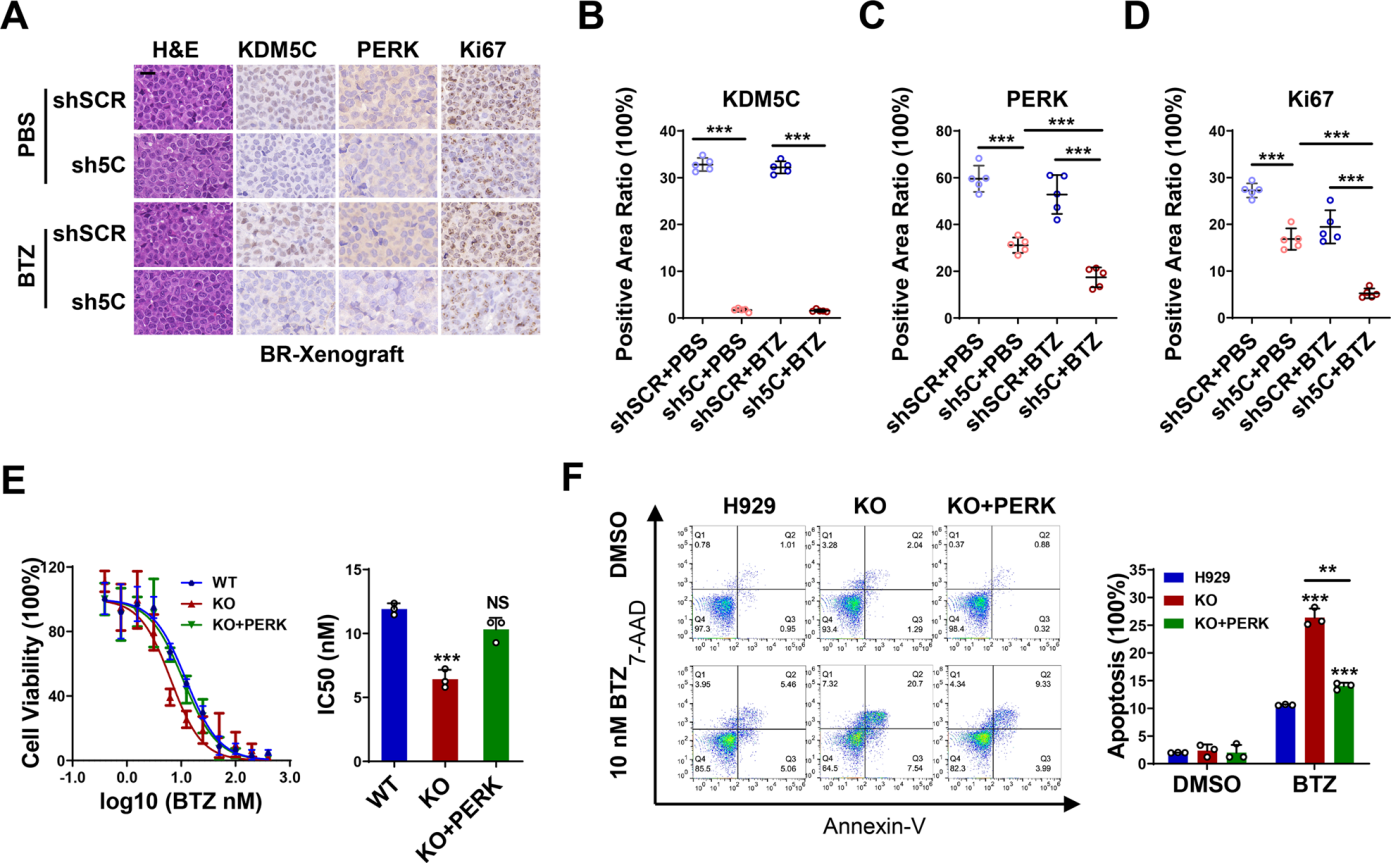
KDM5C confers bortezomib resistance via PERK in MM. **A** IHC results showing protein staining of KDM5C, PERK and Ki67 in KDM5C-knockdown (sh5C) BR cell-derived xenografts compared with shSCR xenografts with or without BTZ treatment. **B**–**D** Quantitative graph of the IHC results shown in (**A**). **E** The IC_50_ of BTZ in PERK-rescued cells (KO + PERK). **F** Apoptosis rates induced by BTZ from FACS of PERK-rescued H929 cells. *P* values were determined by two-way ANOVA (**B**–**D**) and Student’s *t* test (**E**, **F**). **P < 0.01; ***P < 0.001; NS not significant.

We showed that KDM5C activates PERK expression to increase the UPR^ER^. Next, we investigated whether KDM5C contributes to BTZ resistance via PERK induction. To test the effects of KDM5C on the BTZ resistance of MM cells, we examined the expression of PERK and the phosphorylation of Nrf2 (p-Nrf2) in BTZ-resistant (BR) cells and xenograft tissues. As shown in Supplementary Fig. S4J, KDM5C-KD significantly decreased PERK expression and Nrf2 phosphorylation in BR cells. Importantly, this inhibitory effect of KDM5C knockdown on the PERK-p-Nrf2 pathway was maintained even when BR cells were stimulated with BTZ. In addition, we also observed that the expression of PERK was significantly lower in the tumor tissues of KDM5C-KD of BR xenografts than in those of the SCR cells, and that the expression of PERK was much lower in the KDM5C-KD with BTZ treatment group than in the SCR with BTZ treatment group (Fig. 5A–D). These results suggest that PERK may play an important role in KDM5C-induced BTZ resistance.

To further confirm the role of PERK in BTZ resistance in MM, we investigated the BTZ response in KDM5C-knockdown cells exogenously overexpressing PERK. As shown in Fig. 5E, F, forced PERK expression in KDM5C-KO cells significantly reversed the reduction in the IC50 value and the increase in the apoptosis rate resulting from BTZ treatment. Collectively, these results indicate that KDM5C confers bortezomib resistance via PERK in MM.

### KDM5C forms a novel complex with CBP and MYC in MM cells

**Fig. 6:**
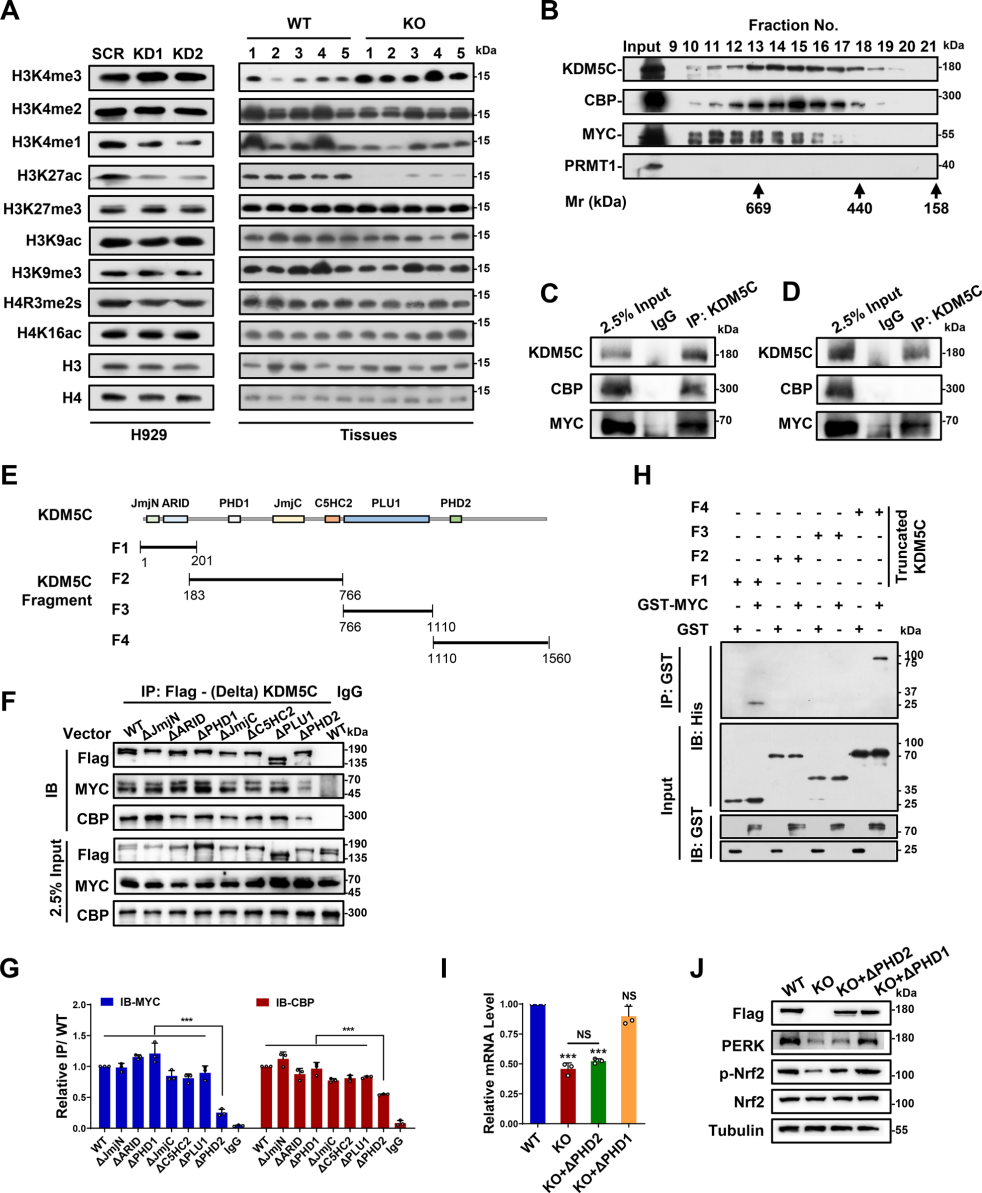
KDM5C forms a novel complex with MYC and CBP in MM cells. **A** Global histone modifications of KDM5C-knockdown (KD) cells and xenograft tumor tissues. **B** Cofractionation of KDM5C and the MYC/CBP complex by fast protein liquid chromatography (FPLC). **C** Co-IP of KDM5C with MYC and CBP in H929 nuclear extractions. **D** Western blots of the endogenous KDM5C, MYC and CBP immunopurified complexes. Co-IP beads were washed with high-salt buffer (500 mM) for three times. Rabbit IgG served as the negative control. **E** Schematic overview of the truncated KDM5C used in the GST pull-down assays in vitro and Co-IP in cells. **F** Co-IP of various indicated KDM5C domain deletion mutants with MYC and CBP in HEK293T cells. **G** Quantitative grayscale analysis of relative IP signals normalized to those of the wild-type full-length protein (WT). **H** GST pull-down assays of GST-MYC with His-tagged truncated KDM5C. **I** Relative mRNA levels of PERK in KDM5C-KO H929 cells after the addition of Flag-tagged KDM5C (WT), ΔPHD2 (KO + ΔPHD2) or ΔPHD1 (KO + ΔPHD1). **J** Protein levels of PERK and Nrf2 in KDM5C-KO H929 cells after the addition of Flag-tagged KDM5C (WT), ΔPHD2 (KO + ΔPHD2) or ΔPHD1 (KO + ΔPHD1). *P* values were determined by Student’s *t* test (H) and one-way ANOVA with Tukey’s multiple-comparison test (**J**). ***P < 0.001; NS not significant.

Next, we explored the mechanism by which KDM5C activates PERK expression to regulate the UPR^ER^. Since KDM5C is a histone H3 lysine 4 dimethyl- and trimethyl-specific demethylase, we first decided to examine the effects of KDM5C knockdown on several key histone markers. As shown in Fig. 6A, KDM5C knockdown slightly decreased global H3K4me1 levels and increased H3K4me3, while significantly reducing H3K27ac level in both H929 cells and H929-KO xenograft tumors tissues (Fig. 6A). All other histone markers were not substantially altered between KDM5C-KD and SCR in H929 cells or H929-KO xenograft tumor tissues (Fig. 6A). Thus, we focused on the possible role of KDM5C in H3K27ac modification. Owing to the global effect of KDM5C on H3K27ac level changes, we hypothesized that protein‒protein interactions might mediate this event.

To test whether KDM5C forms a multiprotein complex in H929 cells in vivo, we performed gel filtration chromatography (GFC) with an H929 nuclear. We analyzed the fraction No. 15 sample collected from the GFC via mass spectrometry (MS) and found that CBP, which triggers H3K27ac [29], was among the proteins in the list (Fig. 6B and Supplementary Table S4). Interestingly, we found that MYC was also present in the list (Fig. 6B and Supplementary Table S4). We further checked the fractions by Western blot and observed that KDM5C was eluted from the earliest protein-containing fractions, defining it as a component of a complex with a molecular mass of approximately 1000 kDa (Fig. 6B). The KDM5C-associated proteins CBP and MYC exhibited peak levels in the fractions containing KDM5C (Fig. 6B). In contrast, PRMT1 tested negative in the same fraction (Fig. 6B). Moreover, KDM5C was confirmed to be able to coimmunoprecipitate with CBP and MYC in H929 nuclear extractions (Fig. 6C). Next, we sought to clarify whether the interactions between KDM5C, CBP, and MYC are direct or indirect. We performed co-IP experiments using a high-salt wash buffer (500 mM NaCl) to minimize non-specific indirect protein interactions—a strategy validated in previous studies [30, 31]. Under these conditions, the strongest retained interactions are indicative of direct binding. As shown in Fig. 6D, CBP was not co-immunoprecipitated with KDM5C after high-salt washing, whereas MYC was still efficiently pulled down with KDM5C. These results demonstrate that KDM5C interacts directly with MYC but indirectly with CBP.

In addition, the direct interaction between KDM5C and MYC was further demonstrated by a GST pull-down assay (Fig. 6E, H). We showed that the F4 region (aa1110-1560) of KDM5C, which contains the second PHD (PHD2), strongly interacts with GST-tagged MYC, whereas the F1 region (aa1-201) of KDM5C, which contains the JmjN and ARID domains, interacts weakly with MYC in vitro (Fig. 6E, H). To further confirm which part/domain of KDM5C contributes to the interaction with MYC or CBP in cells, we constructed various Flag-tagged KDM5C expression cassettes with the individual domains deleted (Fig. 6E, F). We showed that the interaction of MYC and CBP with KDM5C was greatly reduced after PHD2 was deleted from KDM5C (ΔPHD2), which is consistent with the results of the GST pull-down (Fig. 6E, F, G, H). In contrast, we observed similar interactions of MYC and CBP with domain deletion KDM5C mutants as with the wild type (WT), indicating that the PHD2 domain in KDM5C is key for the interaction of MYC-CBP with KDM5C (Fig. 6E, F, G). Notably, this binding mechanism differs from a previous report by Shen et al. [9], in which ERα was shown to directly bind KDM5C and mask its enzymatic active site. In fact, we found that the ΔPHD2 deletion mutant KDM5C was not able to rescue the expression of PERK and phosphorylation of Nrf2 when expressed in KDM5C knockout H929 cells, whereas the ΔPHD1 deletion mutant KDM5C was able to rescue the expression of PERK and phosphorylation of Nrf2 (Fig. 6I, J). Taken together, these results indicate that KDM5C forms a novel complex with CBP and MYC in which the PHD2 domain is crucial for their interaction in MM cells.

### The KDM5C-CBP-MYC complex facilitates H3K27ac to modulate PERK transcription

**Fig. 7:**
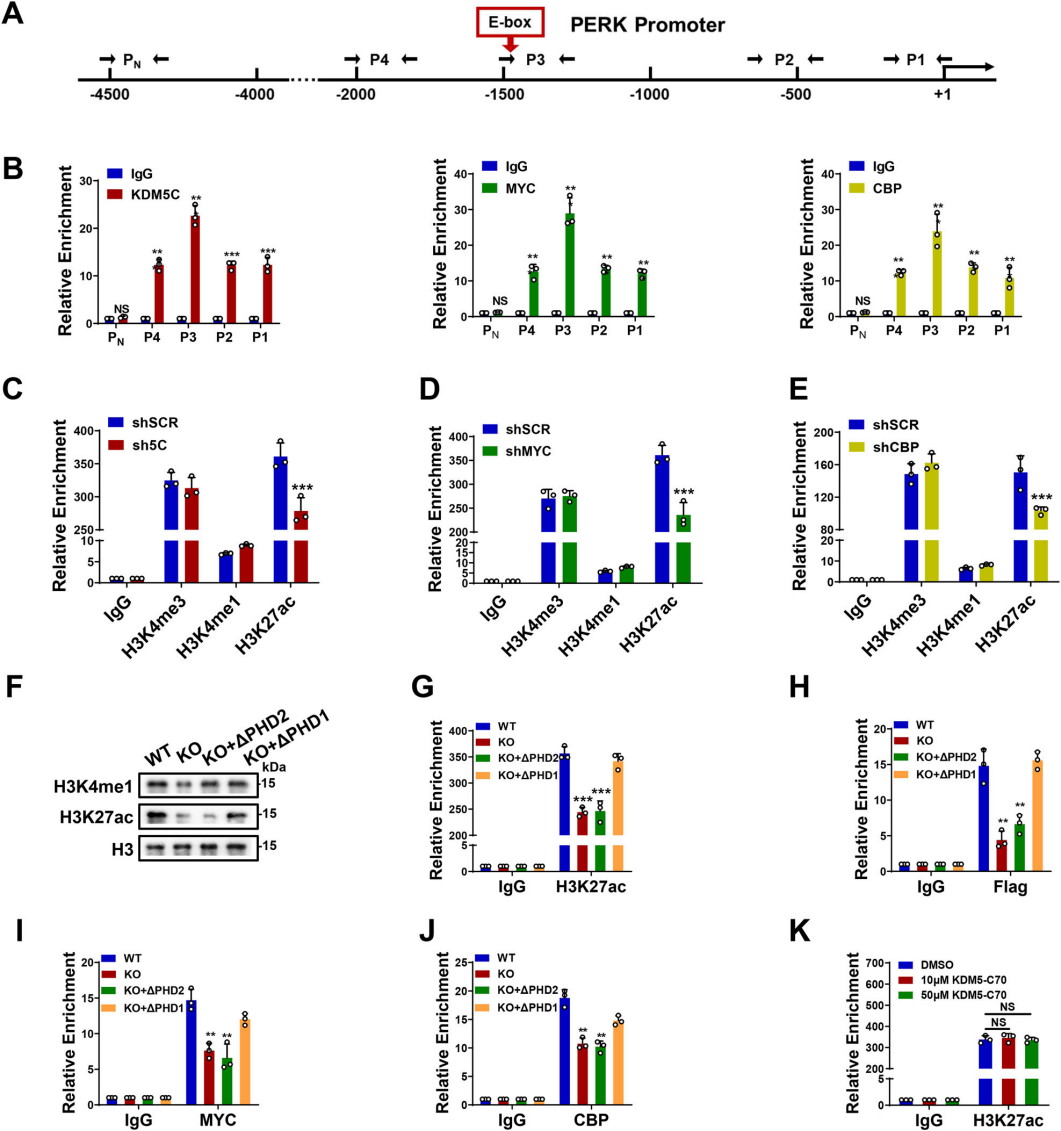
The KDM5C-MYC-CBP complex facilitates H3K27ac at the promoter to modulate PERK transcription. **A** Schematic overview of 5 pairs of walking primers on the PERK promoter; P_N_ was used as the negative control. **B** ChIP‒qPCR analysis of KDM5C, MYC, and CBP enrichment at the PERK promoter. ChIP‒qPCR of H3K4me3/H3K4me1/H3K27ac enrichment on the PERK promoter after knocking down KDM5C (**C**), MYC (**D**) and CBP (**E**). **F** Global histone modifications in KDM5C-KO H929 cells after the reconstruction of ΔPHD2 and ΔPHD1. **G** ChIP‒qPCR analysis of H3K27ac enrichment at the PERK promoter in KDM5C-KO H929 cells after recognition by Flag-tagged KDM5C (WT), ΔPHD2 (KO + ΔPHD2) or ΔPHD1 (KO + ΔPHD1) was performed. ChIP‒qPCR of Flag-tagged KDM5C/Flag-tagged ΔPHD2 and ΔPHD1 (**H**), MYC (**I**), and CBP (**J**) enrichment of the PERK promoter in KDM5C-KO H929 cells after recognition of Flag-tagged KDM5C (WT), ΔPHD2 (KO + ΔPHD2) or ΔPHD1 (KO + ΔPHD1) was performed. **K** ChIP‒qPCR analysis of H3K27ac enrichment at the PERK promoter in H929 after KDM5 inhibitor, KDM5-C70 treatment for 48 h*. P* values were determined by Student’s *t* test or one-way ANOVA with Tukey’s multiple-comparison test (**G**–**J**). **P < 0.01; ***P < 0.001.

To validate the role of the KDM5C-CBP-MYC complex in PERK transcription, we conducted ChIP-qPCR using six primer pairs targeting the PERK promoter. We found that KDM5C/CBP/MYC was significantly enriched with a similar pattern at the PERK promoter (Fig. 7A, B). Interestingly, we observed that the presence of a MYC-binding E-box motif (5’-CACGTG-3’) coincided with the ChIP peak P3 signals (Fig. 7A, B). To our surprise, the existence of the KDM5C-CBP-MYC complex at the PERK promoter is mutually dependent, as shown by the ChIP analysis of H929 cells with individual knockdown (Supplementary Fig. S5A–C).

Given that CBP is closely related to histone H3K27ac and KDM5C is highly associated with histone H3K4me1/3 marks, we conducted ChIP assays to explore the alterations in H3K27ac or H3K4me1/3 enrichment at the PERK promoter region (P3) following the knockdown of KDM5C/MYC/CBP in H929 cells. Results showed significantly reduced H3K27ac enrichment at the PERK promoter in KDM5C/MYC/CBP-knockdown cells compared to SCR cells (Fig. 7C–E). In contrast, H3K4me1/3 enrichment at the PERK promoter was unchanged in the KDM5C/MYC/CBP-knockdown cells compared with the SCR cells. Consistently, knocking down MYC or CBP significantly reduced PERK expression and the phosphorylation of Nrf2 in H929 cells (Supplementary Fig. S5D–G). Importantly, H3K27ac levels could not be rescued by forced overexpression of the ΔPHD2 deletion mutant KDM5C in KDM5C-knockout H929 cells, whereas forced overexpression of the ΔPHD1 deletion mutant KDM5C in KDM5C-knockout H929 cells rescued H3K27ac levels (Fig. 7F). Consistent results with H3K27ac enrichment were obtained via ChIP assays, in which the ΔPHD1/ΔPHD2 deletion mutants KDM5C were forced overexpressed in KDM5C-knockout H929 cells (Fig. 7G). We confirmed that the enrichment of KDM5C, CBP or MYC from the ΔPHD2 deletion mutant KDM5C-CBP-MYC complex was significantly reduced, whereas that from the ΔPHD1 deletion mutant KDM5C-CBP-MYC complex was similar to that of the WT (Fig. 7H–J).

To further establish the functional mechanism by which KDM5C and MYC regulate PERK transcription, we constructed luciferase reporter plasmids containing either the wild-type (WT) PERK promoter or a mutant PERK promoter with a disrupted MYC-binding E-box. These plasmids were transfected into HEK293T cells, and luciferase activity was measured in both scramble control (SCR) and KDM5C-knockdown (KD) cells. As shown in Supplementary Fig. S5H, E-box mutation alone significantly reduced PERK promoter-driven luciferase activity; importantly, simultaneous KDM5C knockdown and E-box mutation resulted in a further significant decrease in luciferase activity. These results confirm that PERK promoter activity is co-regulated by both MYC (via the E-box) and KDM5C.

To further confirm whether KDM5C exerts its effect via its enzymatic activity in this model, we treated MM cells with the KDM5 inhibitor KDM5-C70 [32] and evaluated its impact on PERK expression and promoter activity. The results showed that KDM5-C70 treatment did not alter H3K27ac levels at the PERK promoter or the protein levels of PERK and Nrf2 in MM cells (Fig. 7K and Supplementary Fig. S5I), indicating that KDM5C regulates PERK expression independently of its enzymatic activity.

Taken together, these results suggest that the KDM5C-CBP-MYC complex modulates PERK expression through CBP-mediated H3K27ac rather than through the canonical demethylase activity of KDM5C.

## Discussion

Bortezomib (BTZ) resistance poses substantial hurdles in clinical practice. Here, we demonstrate that KDM5C contributes to BTZ resistance in MM by forming a novel complex with CBP and MYC. This complex augments the UPR^ER^ through the PERK‒Nrf2 axis, suggesting a novel therapeutic target for BTZ resistance in MM.

KDM5C has been demonstrated to play intricate roles within tumors. It exhibits abnormal expression patterns in many tumors and governs various biological functions, such as tumor cell proliferation [9, 12, 13, 33–35], tumor immunity [36] and resistance to multiple drugs [15, 17, 37–39]. Our findings align with the effect of KDM5-C70, a pan-inhibitor of the KDM5 family, which curtails the proliferation of MM cells in vitro [32]. Specifically, our study revealed that KDM5C modulates MM growth and BTZ resistance via the PERK‒Nrf2 axis within the UPR^ER^. As reported, PERK is highly expressed in multidrug-resistant cells. In HT29 chemotherapeutic-resistant cancer cells, PERK expression is significantly elevated, and PERK knockdown suppresses MRP1 expression and restores drug sensitivity [40]. Notably, the role of PERK in MM appears to be twofold; that is, either the inhibition or activation of PERK can impede MM cell proliferation and trigger apoptosis [41–44]. A study showed that the knockdown of PERK has been shown to reduce cell survival and induce apoptosis in MM cells [44], suggesting that the increased PERK basal expression levels in MM cells are essential for their survival and proliferation. On the other hand, the activation of PERK can also lead to growth arrest and/or apoptosis, as it has been linked to the induction of genes such as the transcription factor GADD153/CHOP [43] or inhibition of cyclin D1 [45]. Thus, inhibiting or activating PERK significantly impacts MM cell proliferation and apoptosis. Previous reports suggested that PERK activation might confer chemotherapy resistance via an Nrf2-dependent pathway [40]. In line with this, Nrf2 phosphorylation regulates cell proliferation, enhances antioxidant capacity, facilitates drug efflux, and promotes cancer cell survival [46]. Interestingly, the PERK inhibitor GSK2606414 augments BTZ cytotoxicity in MM cells [44], corroborating our findings. Overall, our study confirms that KDM5C affects MM cell proliferation and BTZ sensitivity by modulating PERK expression, identifying a potential target for BTZ-resistant MM.

To date, strategies to overcome BTZ resistance have focused on developing inhibitors targeting other proteasomes or key regulators within compensatory pathways, such as MEK and HDAC inhibitors [47]. Indeed, studies show that inhibiting KDM5C in colon cancer enhances sensitivity to oxaliplatin and irinotecan by suppressing ABCC1 [15]. Notably, our study found that KDM5C knockdown significantly reduced ABCC11 expression but not ABCC1, as evidenced by our RNA-sequencing data (PRJNA1062085). Therefore, although our current study suggests that KDM5C is associated with the UPR^ER^ to impart BTZ resistance in MM, we cannot rule out the potential involvement of other drug transport or metabolic pathways linked to KDM5C in contributing to BTZ resistance.

Several studies have also documented the transcriptional activation potential of KDM5C [48, 49]. While KDM5C may regulate transcription independently of its enzymatic activity, as many mutations in XLMR patients occur in nonenzymatic regions [22, 50]. In this study, we revealed a novel transcriptional activation mechanism of KDM5C, which forms an unprecedented complex with CBP and MYC to epigenetically activate gene transcription by mediating H3K27ac on the PERK promoter, accentuating the role of the PHD2 domain of KDM5C as a scaffold. Intriguingly, this regulatory role of KDM5C is decoupled from its canonical demethylase enzymatic activity on H3K4me2/3. Indeed, prior studies have shown that KDM5C can be recruited by MYC in mouse embryonic stem cells, yet it catalyzes the demethylation of H3K4me3 on gene promoters and enhancers, thereby suppressing gene expression [48]. Hence, the role of KDM5C might be contingent upon the cell type or context. In our study, we revealed that KDM5C participates in the transcriptional activation of PERK, which is reciprocally reliant on MYC. Given that MYC is regarded as an “undruggable” target owing to its structural idiosyncrasies, our findings might offer novel perspectives for devising effective therapies against MYC. Since KDM5C primarily confers bortezomib (BTZ) resistance in multiple myeloma via pathways independent of its demethylase activity, targeting its catalytic function alone cannot fully eliminate its ability to support cancer cell survival. Notably, PROTAC-mediated degradation of KDM5C has been reported [51], which presents a unique advantage: it enables the elimination of both KDM5C’s enzymatic and non-enzymatic activities. Consequently, combining a KDM5C-targeted PROTAC with BTZ is expected to amplify proteotoxic stress in MM cells, dampen their adaptive stress response, and delay the development of BTZ resistance. This dual therapeutic strategy merits preclinical investigation and holds substantial translational potential for improving clinical outcomes in patients with BTZ-resistant MM.

In summary, we revealed that KDM5C, which is highly expressed in MM, assembles a novel complex with MYC and CBP. This triggers the accumulation of H3K27ac on the PERK promoter. The transcriptional upregulation of PERK activates the phosphorylation of Nrf2, promoting MM progression and BTZ resistance in MM. This discovery highlights an alternative therapeutic approach for overcoming BTZ resistance in MM.

## Materials and methods

### Cell culture and CD138^+^ primary patient cells isolation

The MM cell line H929 was purchased from ATCC. LP-1 and BTZ-resistant MM cells developed from LP-1 were gifts from Dr. Zhiqiang Liu (Tianjin Medical University, China). The cells were cultured at 37 °C in a humidified incubator with 5% CO_2_. CD138^+^ primary patient cells were isolated from bone marrow via CD138^+^ MicroBeads (STEMCELL Technologies) according to the manufacturer’s instructions. The cells were negative for mycoplasma contamination.

### Generation of knockdown and KDM5C knockout cell lines

ShRNAs targeting KDM5C, MYC, and CBP were cloned and inserted into pLL3.7 or pLKO-TRC vectors, respectively. The target sequences were as follows: shKDM5C-1: 5’- GCTGAGACAGCTAGAGCTA -3’, shKDM5C-2: 5’- GGCGGATCTTGGACCTCTA -3, shMYC-1: 5’- CCTGAGACAGATCAGCAACAA -3’, shMYC-2: 5’- CAGTTGAAACACAAACTTGAA -3’, shCBP-1: 5’- CCCGATAACTTTGTGATGTTT -3’, shCBP-2: 5’- ATCGCCACGTCCCTTAGTAAC -3’. The KDM5C knockout plasmid was constructed with px458 (sgKDM5C: 5’- CGGTGGCGGTAGGAAATCGT -3’).

### Transfection, virus packaging and infection

siRNAs were synthesized by Shanghai GenePharma. The targeting sequences were as follows: siKDM5A: 5’- CCTTGAAAGAAGCCTTACAAA -3’, siKDM5B: 5’- GUGCCUGUUUACCGAACUAAU -3’, siKDM5C: 5’- GGCGGAUCUUGGACCUCUA -3’. siRNA and plasmid transfection were performed via Lipofectamine 3000 (Life Technologies) according to the manufacturer’s protocol.

### Tumor xenograft experiments

The sample size was chosen with adequate power on the basis of the literature and our previous experience [52]. Five-week-old female NSG mice (GemPharmatech, Nanjing) were used to establish xenografts. For the drug resistance model, BR MM cells were injected subcutaneously into mice. After the tumor volume reached 50 mm^3^, the mice were treated with BTZ (1 mg/kg) (TargetMol, Shanghai) or PBS every 3 days. The mice were weighed, and the tumors were measured every 3 days. The ethics committee has set the maximum tumor size limit as 2000 mm³. Throughout the course of our study, the tumor size of all animals remained within the permitted range as defined by the ethics committee.

### RNA isolation and real-time qPCR

The total RNA used for RT‒qPCR was purified with TRIzol reagent and reverse transcribed using HiScript III All-in-one RT SuperMix (Vazyme, Nanjing). Real-time qPCR was performed via AceQ qPCR SYBR Green Master Mix (Vazyme, Nanjing) in a C1000 PCR System (Bio-Rad, USA). Fold changes were calculated via the 2-ΔΔCt method, with ACTB as a reference. The primers are listed in Supplementary Table S1.

### Western blotting

Western blotting was performed as described previously [53]. Briefly, protein lysates were prepared in lysis buffer. The cell lysates were separated via SDS‒PAGE and transferred to PVDF membranes. After blocking, the membranes were incubated and infiltrated overnight at 4 °C with specific antibodies. The next day, incubated with secondary antibodies. The signals were detected with an enhanced chemiluminescence kit (Tanon, Shanghai). The antibodies are listed in Supplementary Table S2.

### Protein purification and in vitro pull-down assays

The fused glutathione-S-transferase (GST) or His tag protein was expressed in *Escherichia coli* BL21 (DE3), the cells were harvested and sonicated, the protein suspension was subjected to affinity chromatography and eluted. For the in vitro pull-down assay, the purified proteins were mixed in equal amounts, incubated at 4 °C for 2 h, combined with glutathione beads (GenScript Biotech, Nanjing), and detected by western blot analysis.

### Gel filtration chromatography (GFC)

H929 nuclear protein was extracted and dialyzed against lysis buffer (20 mM HEPES, 300 mM NaCl, 0.1 mM EDTA, pH 8.0). A total of 5 mg of nuclear protein was concentrated to 1 ml via a Millipore Ultrafree centrifugal filter apparatus (10 kDa nominal molecular mass limit) and then separated via a Superose 6 size exclusion column (Amersham Biosciences). The fractions were collected separately, followed by western blotting and mass spectrometry.

### Chromatin-immunoprecipitation (ChIP)

The ChIP assay was performed as described previously [53]. Briefly, 1 × 10^7^ cells were crosslinked with 1% formaldehyde. Then the nuclear were isolated and resuspended in ChIP lysis buffer for sonication. Chromatin was fragmented into 200–500 bp fragments via Bioruptor Plus (Diagenode, Belgium). For each IP, chromatin was immunoprecipitated with 2–5 mg of antibody in IP dilution buffer at 4 °C overnight. Then, the antibody‒DNA complexes were harvested with protein A/G agarose beads (GenScript Biotech, Nanjing), and the DNA was purified via spin columns. The antibodies and primers are listed in Supplementary Table S3.

### Luciferase reporter assay

The luciferase reporter assay was performed as previously described [54]. Briefly, luciferase reporter vectors were constructed using pGL3 plasmids, which contained either the wild-type PERK promoter (positions –2000 to +1) or a mutant version of this promoter (with a mutation at positions –1504 to –1487: 5’-AAAGACTTTTTTGTAGGA-3’). HEK293T cells were seeded in 12-well plates and co-transfected with the luciferase reporter plasmid and pRL-TK (a Renilla luciferase-containing control reporter plasmid). At 48 h post-transfection, cells were harvested, and luciferase activity was measured using the Dual-Luciferase Reporter Assay System (Vazyme, Nanjing) following the manufacturer’s protocol. Firefly luciferase activity was normalized to Renilla luciferase activity (from pRL-TK) to account for transfection efficiency.

### RNA-Seq and analysis

Total RNA from cells was isolated via TRIzol (Invitrogen, Thermo Fisher Scientific). Library construction and sequencing were performed by Majorbio (Shanghai, China). Data analysis was performed via the Majorbio biological cloud platform (https://cloud.majorbio.com/page/tools/).

### Statistical analysis

The data are presented as the means ± standard deviations (SDs) from at least three independent experiments. Statistical analysis of differences between groups was conducted using Student’s *t* test for pairwise comparisons, one-way ANOVA with Tukey’s post-hoc test for multiple comparisons within a single factor, and two-way ANOVA for interactions between two factors. Survival analysis was performed using Pearson’s chi-squared test and the log-rank test, both conducted with GraphPad Prism 9 software. The IC50 values were determined using nonlinear regression analysis within GraphPad Prism 9. A *P*-value of less than 0.05 was considered to indicate statistical significance compared to the control groups.

## Supplementary information

Supplementary file for: Noncanonical Role of KDM5C in Conferring Bortezomib Resistance via the PERK‒Nrf2 Axis in Multiple Myeloma

Original experimental data

## Data Availability

The datasets generated and analyzed during the current study are available in the Sequence Read Archive (SRA) database, [PRJNA1062085].
