# Supplementary material for: Noncanonical role of KDM5C in conferring bortezomib resistance via the PERK‒Nrf2 axis in multiple myeloma
Source: Cell Death Dis. 2026 Mar 23;17(1):339. doi: 10.1038/s41419-026-08591-7 (PMC13039704; doi:10.1038/s41419-026-08591-7)
Supplement: Supplementary file 1 — Supplementary file for: Noncanonical Role of KDM5C in Conferring Bortezomib Resistance via the PERK‒Nrf2 Axis in Multiple Myeloma [file 41419_2026_8591_MOESM1_ESM.docx]

**Supplementary data for:**

**Noncanonical Role of KDM5C in Conferring Bortezomib Resistance via the PERK‒Nrf2 Axis in Multiple Myeloma**

Peifen Lu^1,2^, Wenbin Shangguan^2^, Weiwei Qian^2^, Dongliang Wu^2^, Wenyang Li^2^, Jingjing Huang^3^, Peipei Xu^2^, Dijun Chen^2^, Feng Li^4^, Bing Chen^2,*^ and Quan Zhao^2,*^

This file includes:

Supplementary Figures S1-5

Supplementary Tables S1-4

**Supplementary Figures**


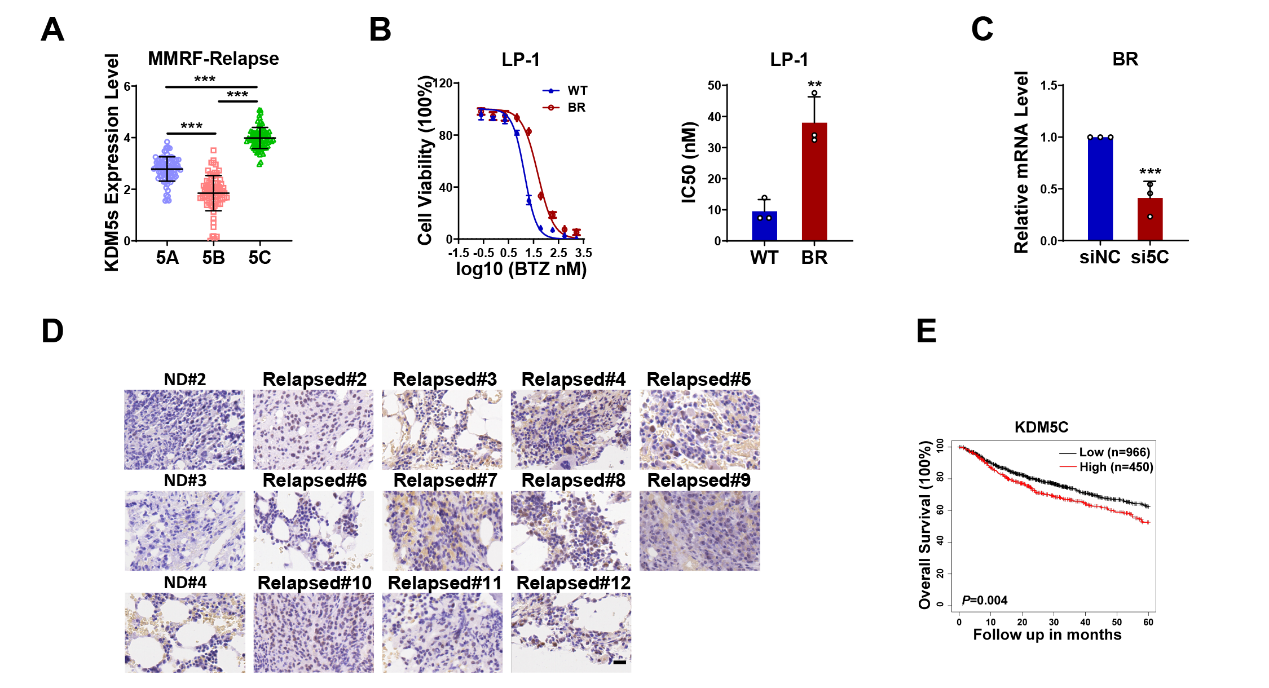


**Fig. S1** Highly expressed KDM5C is associated with poor outcomes and BTZ resistance. **(A)** KDM5A (5A), KDM5B (5B), and KDM5C (5C) expression in MM patients who experienced relapse according to the MMRF CoMMpass datasets. **(B)** The IC_50_ of BTZ in BTZ-resistant cells (BR) cells compared with that in parental LP-1 cells (WT). **(C)** Silencing efficiency of KDM5C (si5C) via siRNA. **(D)** IHC of KDM5C in 3 newly diagnosed (ND) and 11 relapsed MM patients. Scale bar = 20 μm. **(E)** Kaplan–Meier survival analyses of the effect of KDM5C expression on the overall survival of MM patients via the KM plotter analysis tool. *P* values were determined by one-way ANOVA with Tukey’s multiple-comparison test (A), Student’s t test (B and C) and Pearson’s coefficient and log-rank test (E). ** *P* <0.01; *** *P* < 0.001.


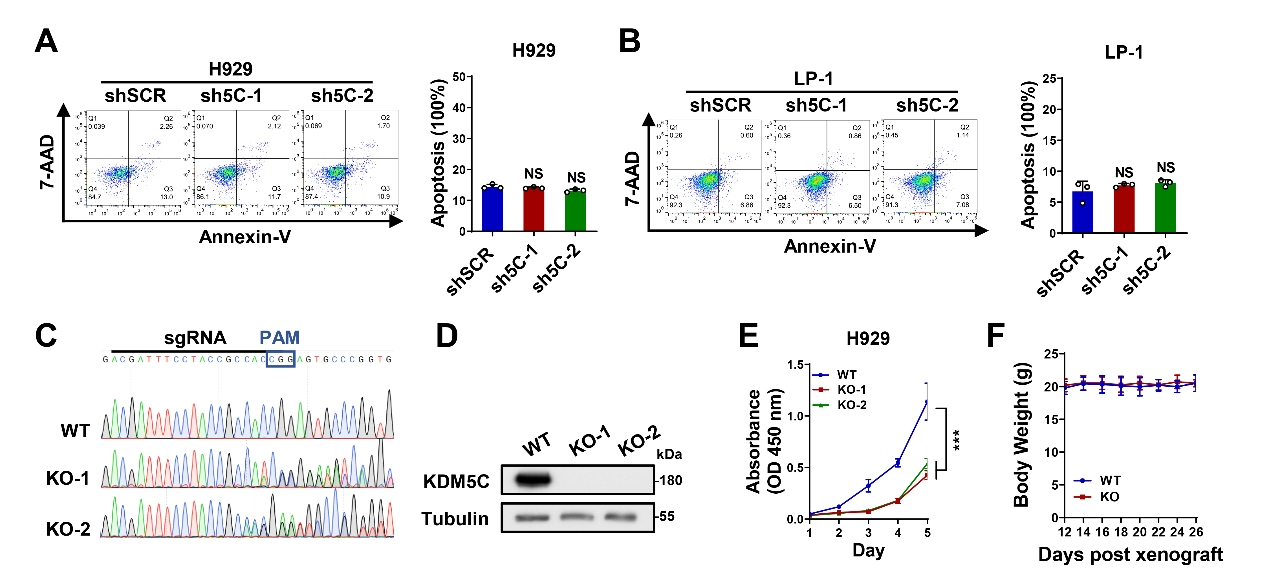


**Fig. S2** KDM5C is required for MM cell proliferation and tumorigenesis. **(A-B)** Apoptosis rate of H929 and LP-1 cells with KDM5C knockdown (sh5C-1, sh5C-2). **(C)** Sequencing results near the PAM of H929 KDM5C-KO (knockout) cell lines. **(D)** KDM5C protein levels in WT and KDM5C-KO cells. **(E)** Growth curve of KDM5C-KO H929 cells. **(F)** Body weights of the mice after subcutaneous injection. *P* values were determined by Student’s t test compared with shSCR/WT. *** *P* < 0.001; NS, not significant.


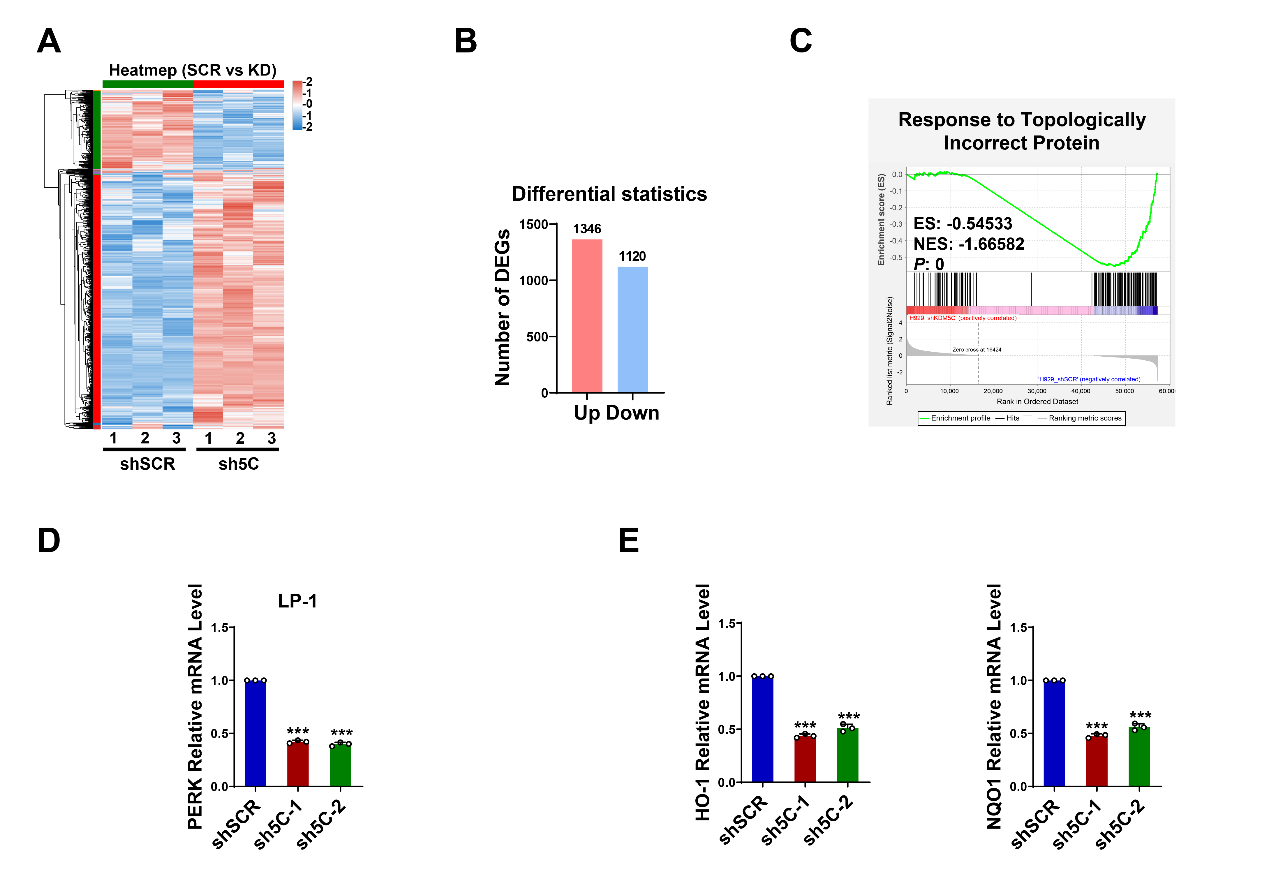


**Fig. S3** KDM5C targeting affects PERK-mediated ER stress in MM. **(A)** Heatmap showing DEGs in the KDM5C-knockdown (sh5C) and control (shSCR) groups by RNA-seq. Up, upregulated genes. Down, downregulated genes. Data from three biological replicates are shown. **(B)** Number of DEGs (differentially expressed genes). **(C)** GSEA enrichment score curves. **(D)** The mRNA level of PERK after knocking down KDM5C (sh5C-1, sh5C-2) relative to the SCR in LP-1 cells. **(E)** The mRNA level of HO-1 and NQO1 after knocking down KDM5C (sh5C-1, sh5C-2) relative to the SCR in H929. ES, enrichment score; NES, normalized enrichment score. *P* values were determined by Student’s t test (D-E) compared with shSCR. *** *P* < 0.001.


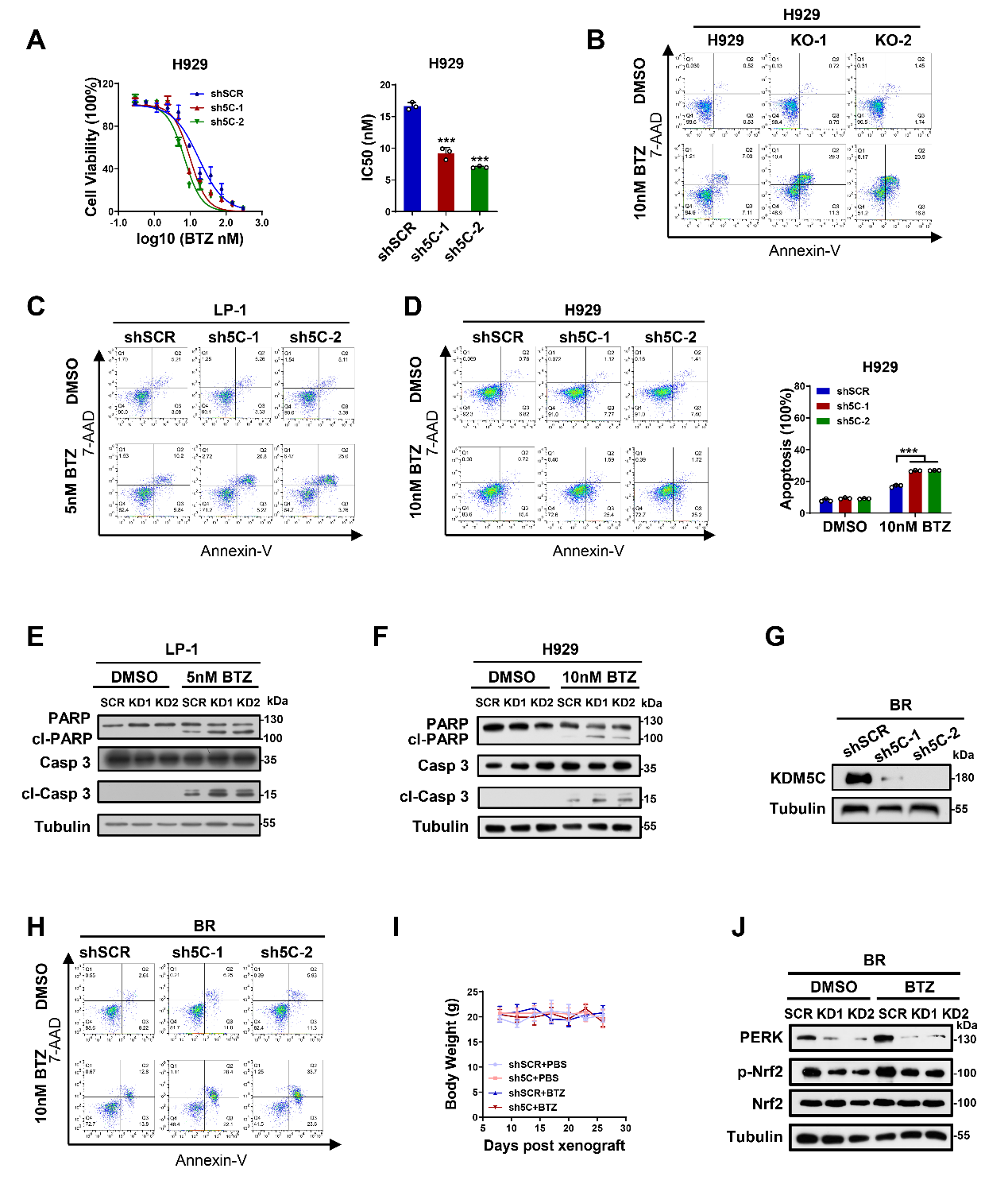


**Fig. S****4** KDM5C promotes the resistance of MM cells to bortezomib. **(A)** The IC_50_ of BTZ in KDM5C-knockdown (sh5C-1, sh5C-2) H929 cells. FACS of apoptosis rates in KDM5C knockout H929 cells **(B).** FACS of apoptosis rates in and LP-1 cells **(C)** and H929 cells with KDM5C knockdown (sh5C-1, sh5C-2) **(D)**. The levels of cleaved PARP and caspase 3 in KDM5C-knockdown (sh5C-1, sh5C-2) LP-1**(E)** and H929 **(F)** cells induced with BTZ. **(G)** The protein level of KDM5C after transduction of the indicated shRNA (sh5C-1, sh5C-2) relative to shSCR in the BR. **(H)** FACS analysis of apoptosis rates in BTZ-resistant (BR) cells with KDM5C knockdown (sh5C-1, sh5C-2). **(I)** Body weights of the mice after subcutaneous injection. **(J)** The level of the PERK-p-Nrf2 pathway after knocking down KDM5C in BR cells after BTZ treatment. BR, bortezomib-resistant. *P* values were determined by Student’s t test (A and D). *** *P* < 0.001.


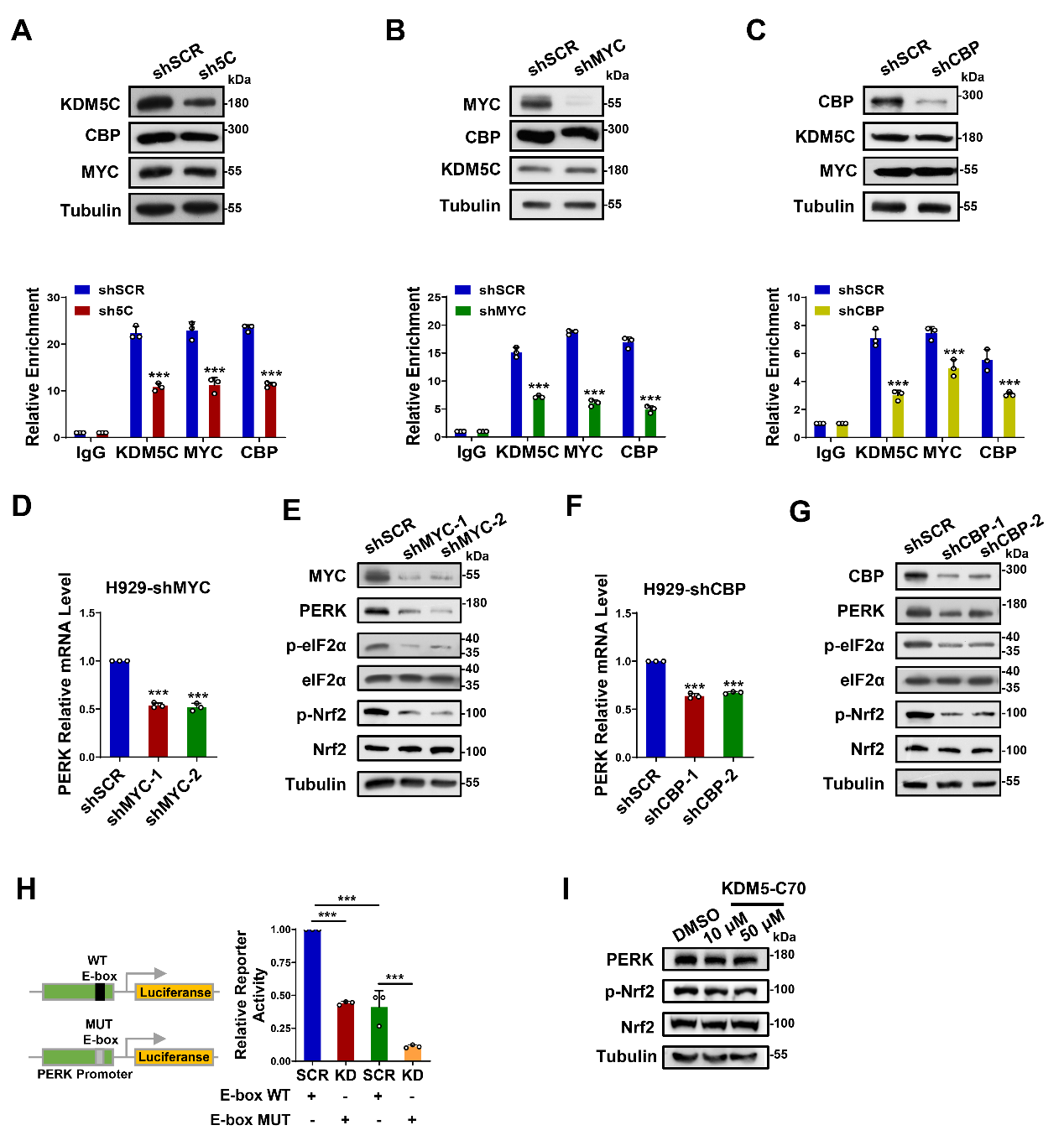


**Fig. S5** The KDM5C-MYC-CBP complex is enriched on the PERK promoter to modulate its transcription. The knockdown efficiency of KDM5C (**A**, upper panel), MYC (**B**, upper panel) and CBP (**C**, upper panel) and their co-enrichment with the PERK promoter (lower panels). **(D)** The mRNA level of PERK in MYC-knockdown cells. **(E)** The level of the PERK-p-Nrf2 pathway after knocking down MYC. **(F)** The mRNA level of PERK in CBP-knockdown cells. **(G)** The level of the PERK-p-Nrf2 pathway after CBP was knocked down. **(H)** Dual Luciferase Assays of PERK. Luciferase activity was corrected for Renilla luciferase activity and normalized to Control group. KD, KDM5C knock down. MUT, mutated on MYC binding motif (E-box). **(I)** The level of the PERK-p-Nrf2 pathway after treating with KDM5 inhibitor KDM5-C70 in 10 μM or 50 μM for 48 h. *P* values were determined by Student’s t test compared with those of IgG (A - C)/shSCR (D, F). *** *P* < 0.001.

**Supplementary Tables:**

**Table S1. The primers used for RT‒qPCR**

| Gene | Sense (5' to 3') | Antisense (5' to 3') |
| --- | --- | --- |
| ACTB | CTCTTCCAGCCTTCCTTCCT | AGCACTGTGTTGGCGTACAG |
| KDM5C | TTGCCAAATTCTGGGAAATC | AGCGTAGCAAGGAGCCAATA |
| KDM5A | GATTTCCGGTGAAGGATGG | CCACGGCACTTTCATACCAG |
| KDM5B | ATTCTGTTGGCACATTGAAGACC | AGCATACCCTGGGACTCCATAC |
| DDIT3 | TTCTCTGGCTTGGCTGACTG | TCCTCCTCTTCCTCCTGAGC |
| WIPI1 | ACTAAAGCCGGGTATAAGCTGT | CGGGATTTCATTGCTTCCGTG |
| ERN1 | CACAGTGACGCTTCCTGAAAC | GCCATCATTAGGATCTGGGAGA |
| SELENOS | ACCTATGGCTGGTACATCGTC | GCCTCAAGGCTCTTAGCCG |
| PERK | ACGATGAGACAGAGTTGCGAC | ATCCAAGGCAGCAATTCTCCC |
| ATF4 | ATGACCGAAATGAGCTTCCTG | GCTGGAGAACCCATGAGGT |
| ERLEC1 | CCGGTGTTACTGGTCCTCTG | AGGGATGTCATCGCTGAGTTG |
| DNAJC18 | TCCACGTATAGTGAGGAACAGC | TTCAGGGCGAGTTTTCTGTAAG |
| TRAF2 | TCCCTGGAGTTGCTACAGC | AGGCGGAGCACAGGTACTT |
| RCN3 | TGTGGCGACCATCAGTTCTG | GTCGTACTGGAAGTTCCCGTG |
| ATF6 | TCCTCGGTCAGTGGACTCTTA | CTTGGGCTGAATTGAAGGTTTTG |
| HO-1 | AAGACTGCGTTCCTGCTCAAC | AAAGCCCTACAGCAACTGTCG |
| NQO1 | GAAGAGCACTGATCGTACTGGC | GGATACTGAAAGTTCGCAGGG |

**Table S2. Primary antibodies**

| **Target** | **Source** | **Manufacturer** | **Cat. No.** |
| --- | --- | --- | --- |
| β-Tubulin | mAb, Mouse | Bioworld | MB65893 |
| KDM5C | pAb , Rabbit | abcam | ab34718 |
| BIP | pAb, Rabbit | Bioworld | BS6479 |
| ATF6α | mAb, Mouse | Santa Cruz | Sc-166659 |
| IRE1α | pAb, Rabbit | HuaBio | ER1902-90 |
| PERK | mAb, Rabbit | CST | 5683 |
| p-eIF2α | mAb, Rabbit | CST | 3398 |
| eIF2α | pAb, Rabbit | proteintech | 11170-1-AP |
| Nrf2 | mAb, Rabbit | Abcam | ab62352 |
| p-Nrf2 (S40) | mAb, Rabbit | Abcam | ab76026 |
| PARP | mAb, Rabbit | CST | 9532 |
| Caspase3 | mAb, Rabbit | CST | 14220 |
| H3K4me3 | pAb, Rabbit | abcam | Ab8580 |
| H3K4me2 | pAb, Rabbit | PTMBIO | PTM-612 |
| H3K4me1 | pAb, Rabbit | abcam | Ab8895 |
| H3K27ac | pAb, Rabbit | ABclonal | A7253 |
| H3K27me3 | mAb, Mouse | abcam | ab6002 |
| H3K9ac | mAb, Mouse | PTMBIO | PTM-156 |
| H3K9me3 | mAb, Mouse | PTMBIO | PTM-743 |
| H4R3me2s | pAb, Rabbit | ABclonal | A3159 |
| H4K16ac | mAb, Mouse | PTMBIO | PTM-187 |
| H3 | pAb, Rabbit | ABclonal | A2348 |
| H4 | pAb, Rabbit | proteintech | 16047-1-AP |
| CBP | mAb, Rabbit | abcam | Ab253202 |
| MYC | pAb, Rabbit | CST | 9402 |
| MYC | mAb, Mouse | abcam | Ab17355 |
| His-tag | mAb, Mouse | Proteintech | 66005-1-Ig |
| GST-tag | mAb, Mouse | Proteintech | 66001-2-Ig |
| Flag-tag | mAb, Mouse | Proteintech | 66008-4-Ig |

**Table S3. The primers used for ChIP‒qPCR**

| Position | Sense (5' to 3') | Antisense (5' to 3') |
| --- | --- | --- |
| P4 | GGCAGGCAAAACAACAAAGG | TAGTGCTGGTCGTAACCCTCAA |
| P3 | CAGCCTGGGCAAAAGAGCA | GCAGAGTGGAGAAGACTGGAATGT |
| P2 | CAATCAAGAGGCAGTTAGCG | TCCCTGGTGGTCAACATCG |
| P1 | CCAGCCTGGGAACATGGAGT | CACCTGAGTGACAGCCTATCTCG |
| P_N_ | TGCTTAGCAGCAGCATGGTCT | TCCTTTTGCCTGTCGTCTCAC |

**Table S4. Components of the nuclear fraction from MS**

| Protein IDs | Unique peptides | Score | Intensity |
| --- | --- | --- | --- |
| Q14839\|CHD4 | 37 | 323.31 | 1651800000 |
| Q92793\|CBP | 24 | 323.31 | 201380000 |
| Q16576\|RBBP7 | 7 | 228.68 | 329400000 |
| O60341\|KDM1A | 16 | 189.48 | 662340000 |
| Q09028\|RBBP4 | 10 | 189.46 | 2296600000 |
| P46821\|MAP1B | 19 | 183.26 | 316320000 |
| Q15061\|WDR43 | 9 | 142.91 | 311010000 |
| Q9NYV4\|CDK12 | 16 | 136.32 | 212820000 |
| O60885\|BRD4 | 16 | 115.03 | 527100000 |
| Q93009\|UBP7 | 15 | 109.31 | 185480000 |
| P19338\|NUCL | 10 | 108.84 | 147180000 |
| Q9BZK7\|TBL1R | 7 | 83.738 | 69790000 |
| Q99856\|ARI3A | 8 | 82.066 | 197660000 |
| Q15910\|EZH2 | 7 | 65.449 | 96137000 |
| Q8WWY3\|PRP31 | 5 | 61.617 | 67321000 |
| P30260\|CDC27 | 8 | 55.724 | 112440000 |
| P61964\|WDR5 | 7 | 50.927 | 91196000 |
| Q9UBL3\|ASH2L | 3 | 47.288 | 82723000 |
| Q6P1J9\|CDC73 | 7 | 46.839 | 98576000 |
| Q08945\|SSRP1 | 6 | 45.058 | 118790000 |
| Q9HCK8\|CHD8 | 6 | 40.749 | 63904000 |
| P41229\|KDM5C | 14 | 37.863 | 299850000 |
| Q9ULU4\|ZMYND8 | 6 | 37.43 | 70093000 |
| Q06587\|RING1 | 3 | 30.998 | 43623000 |
| Q9H9B1\|EHMT1 | 4 | 27.627 | 47097000 |
| P01106\|MYC | 2 | 26.78 | 1847900 |
| Q96B26\|EXOS8 | 4 | 26.093 | 41622000 |
| Q9Y6W5\|WASF2 | 2 | 25.828 | 59156000 |
| Q8NHM5\|KDM2B | 2 | 15.214 | 3066100 |
| O14744\|PRMT5 | 2 | 14.118 | 20426000 |
| P50613\|CDK7 | 1 | 11.256 | 15269000 |
| Q12873\|CHD3 | 1 | 5.9047 | 3849100 |
