# Supplementary material for: Noncanonical role of KDM5C in conferring bortezomib resistance via the PERK‒Nrf2 axis in multiple myeloma
Source: Cell Death Dis. 2026 Mar 23;17(1):339. doi: 10.1038/s41419-026-08591-7 (PMC13039704; doi:10.1038/s41419-026-08591-7)

Original WB data for:

Noncanonical Role of KDM5C in Conferring Bortezomib Resistance via the PERK–Nrf2 Axis in Multiple Myeloma

Peifen Lu[1,2], Wenbin Shangguan[2], Weiwei Qian[2], Dongliang Wu[2], Wenyang Li[2], Jingjing Huang[3], Peipei Xu[2], Dijun Chen[2], Feng Li[4], Bing Chen[2,*] and Quan Zhao[2,*]

Figure 2B

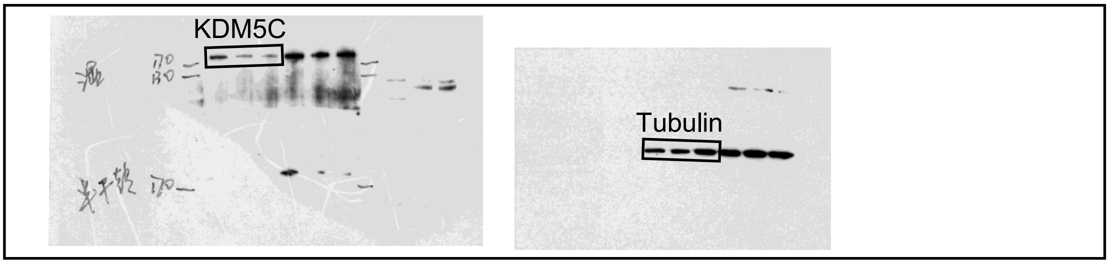

Figure 2E

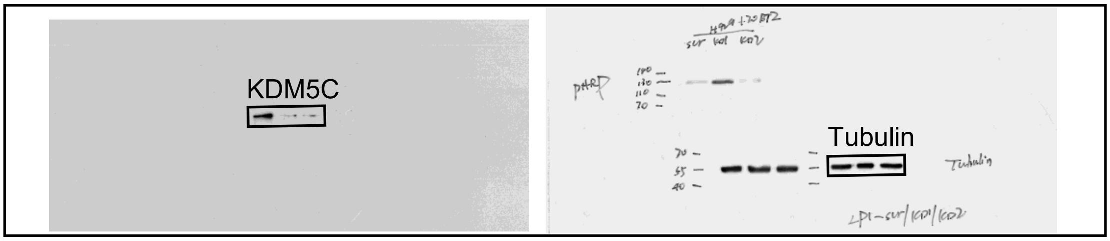

S.Figure 2D

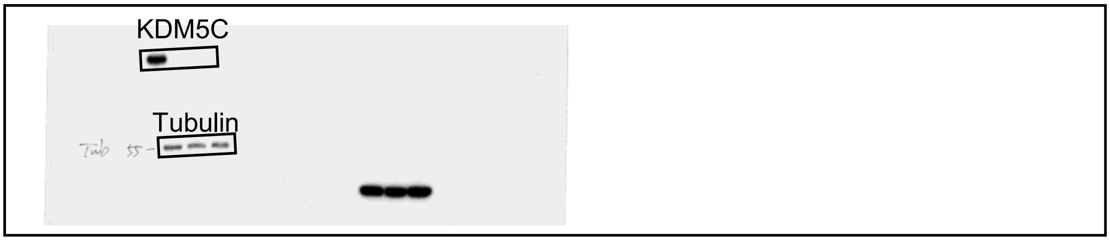

Figure 3E

H929

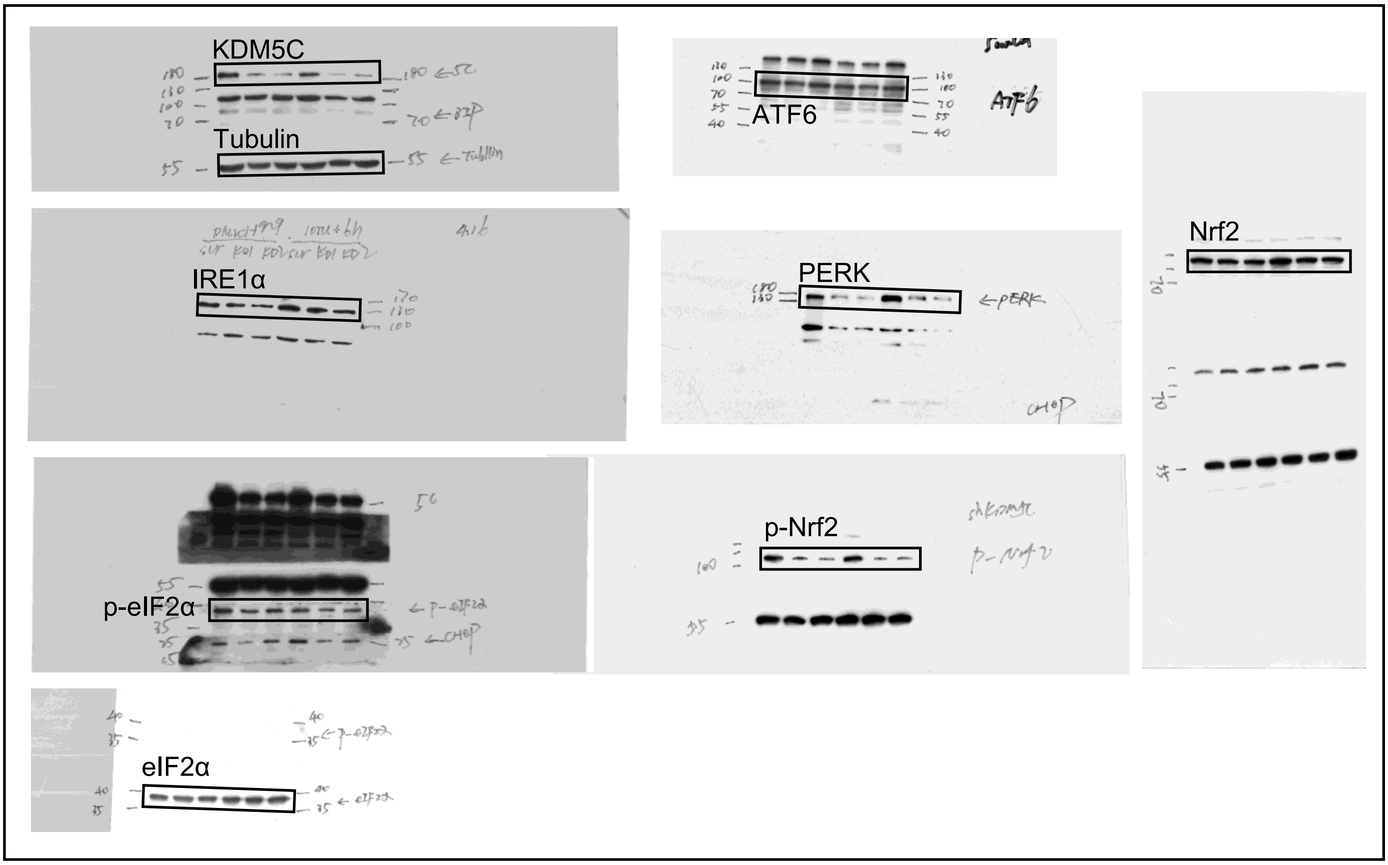

Figure 3E

LP-1

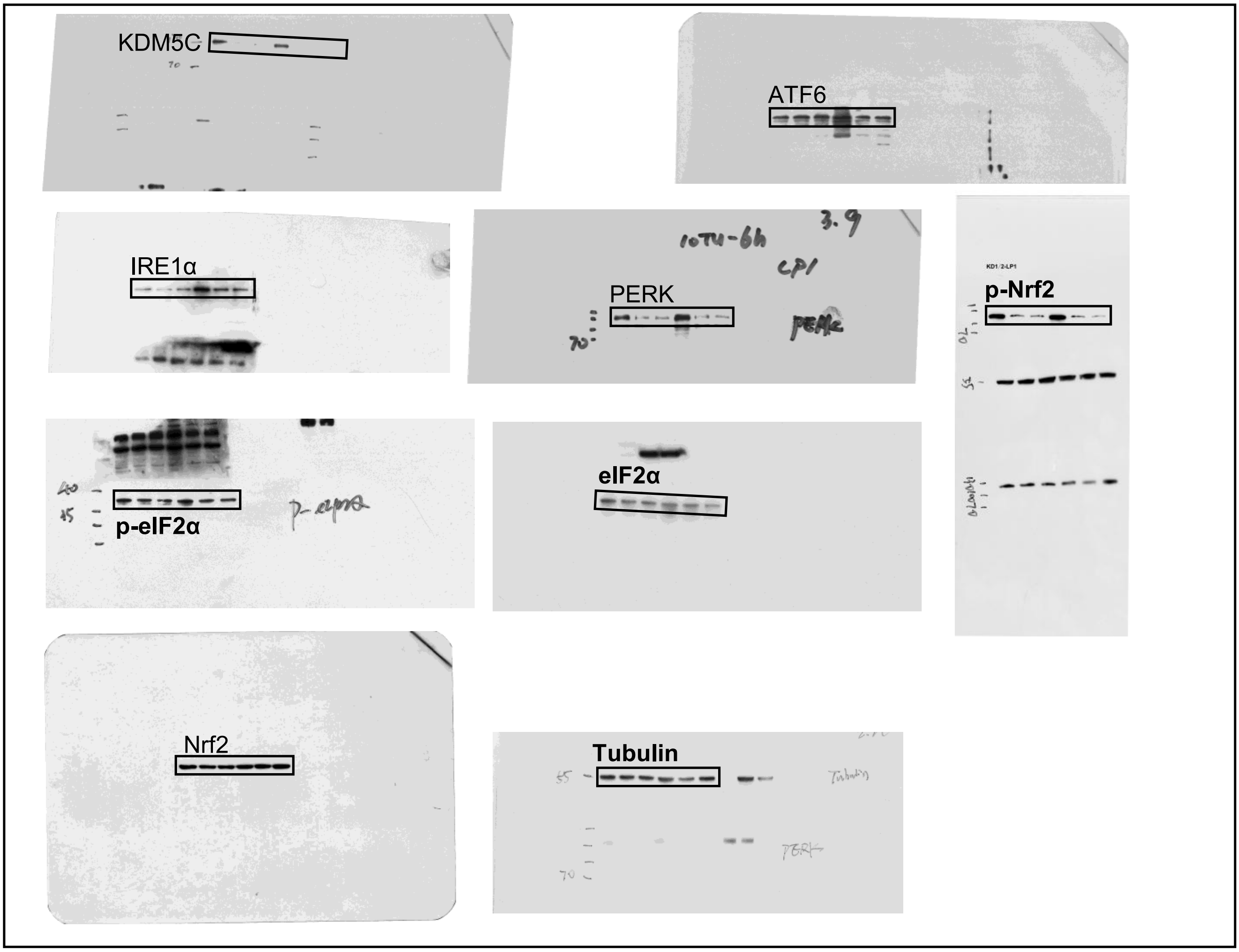

Figure 3J

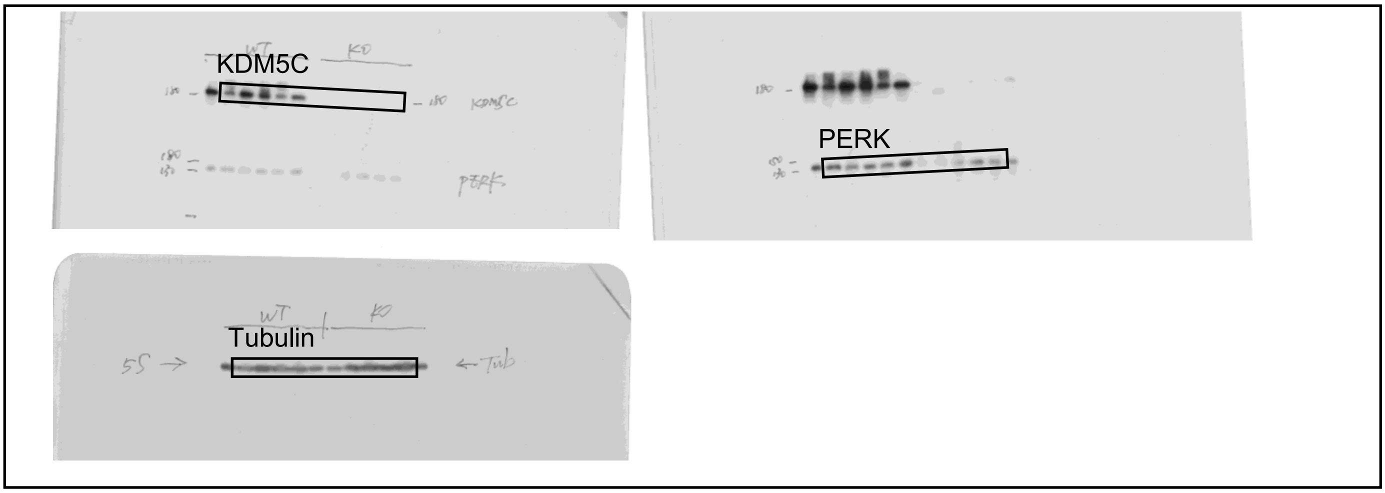

Figure 3K

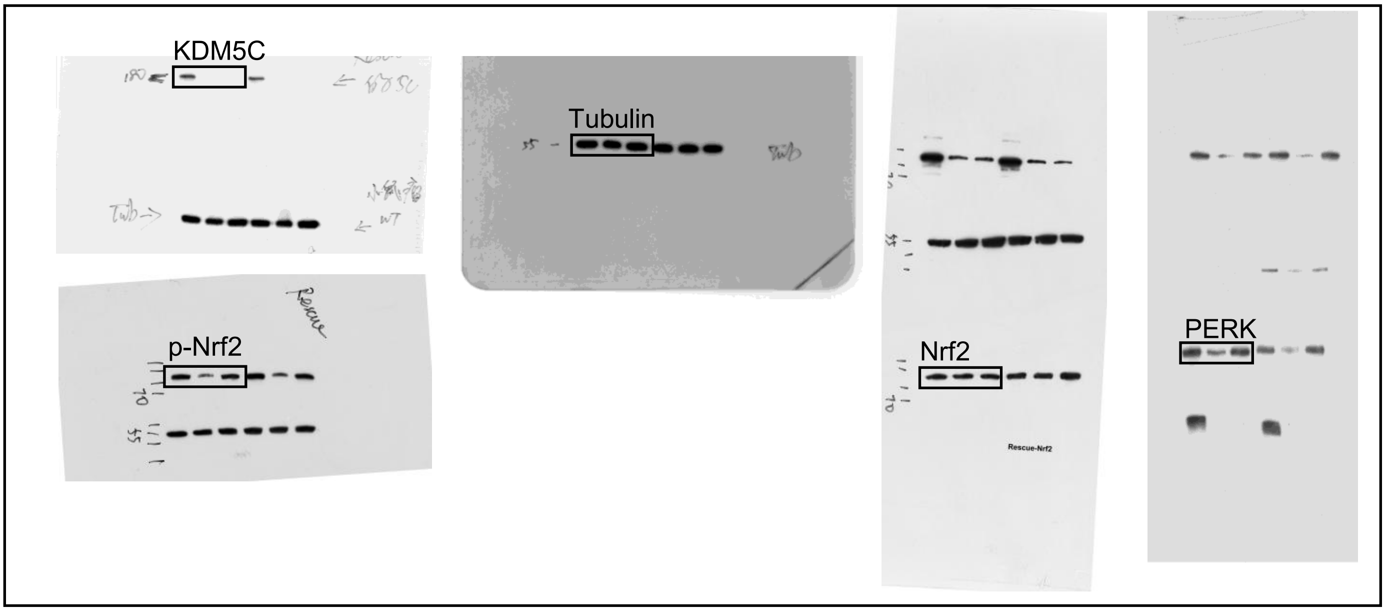

Figure 4F

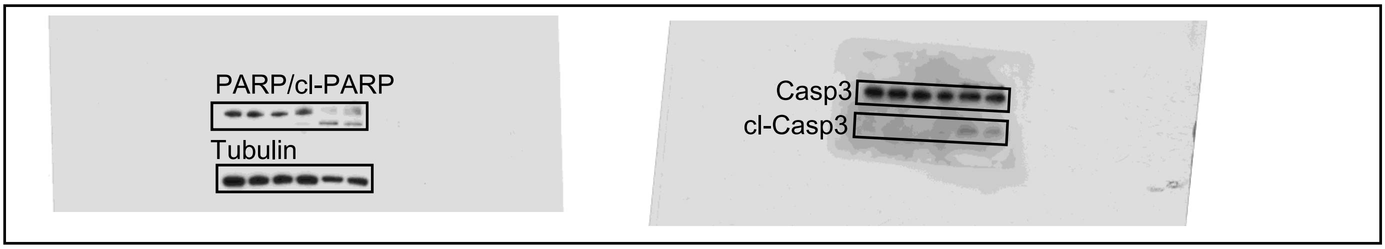

Figure S4E

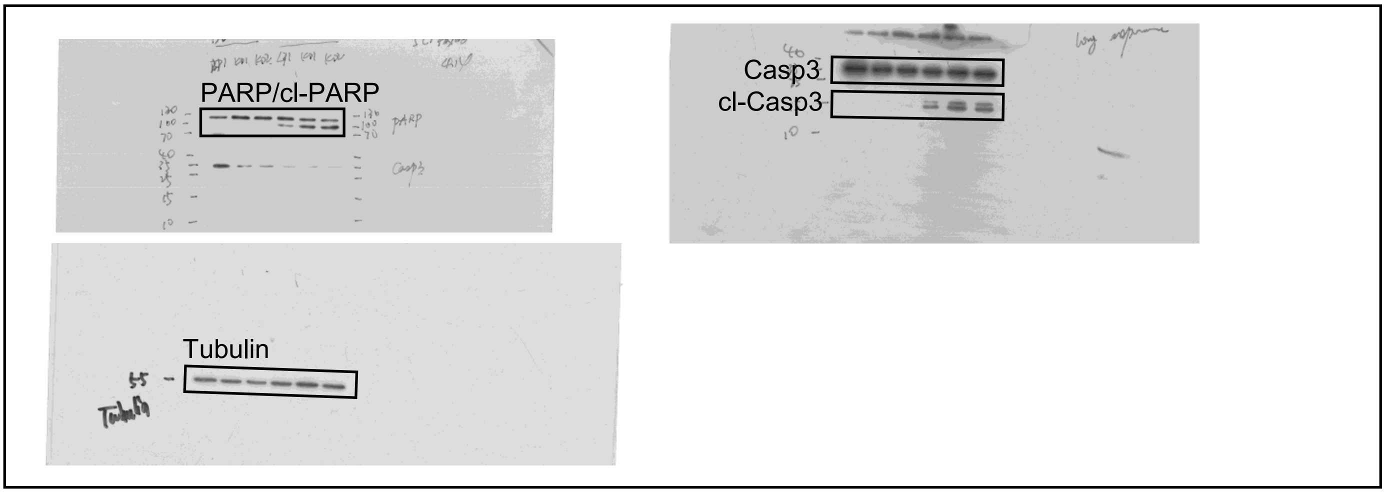

Figure S4F

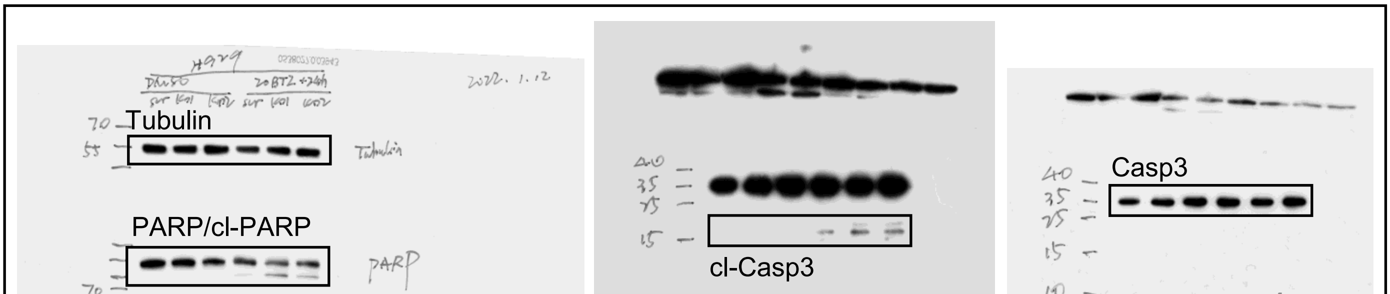

Figure S4G

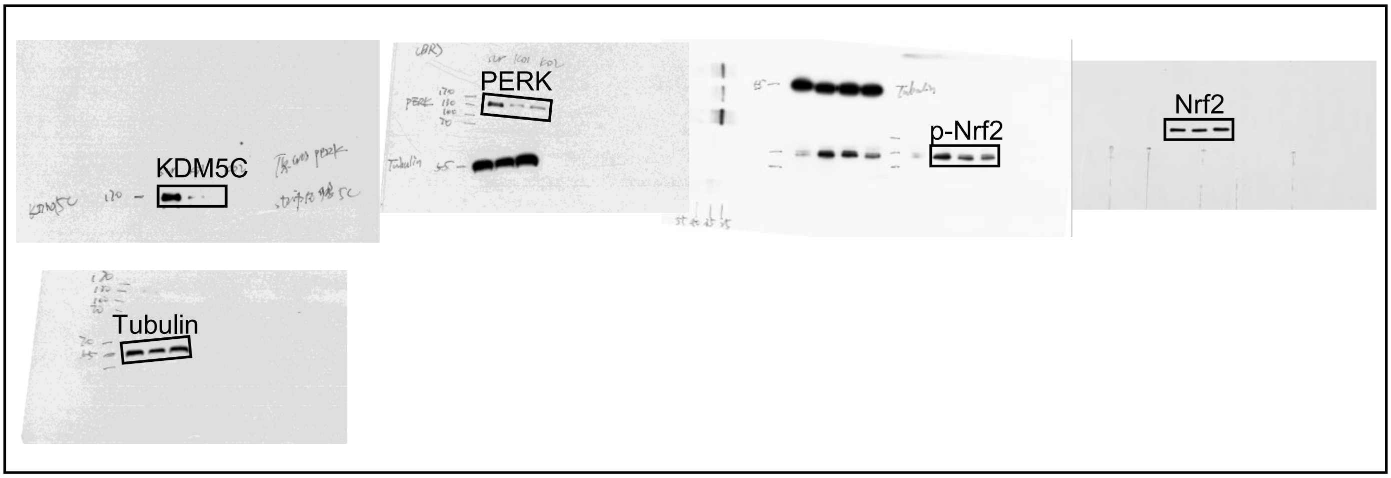

Figure S4J

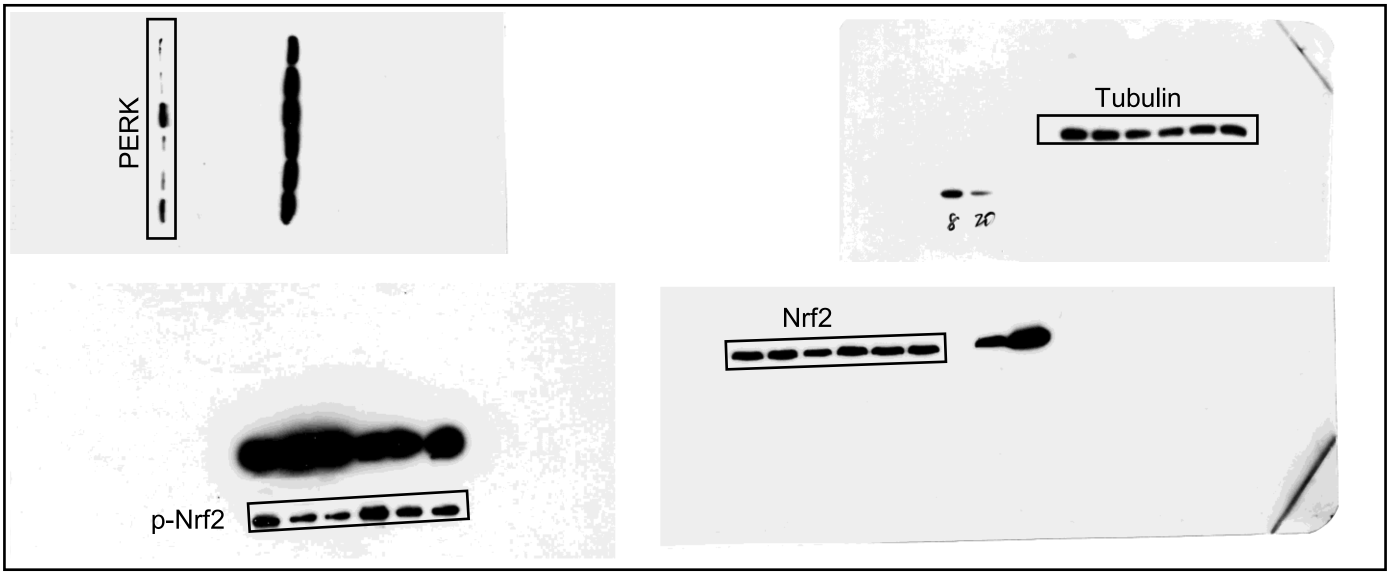

Figure 6A

H929

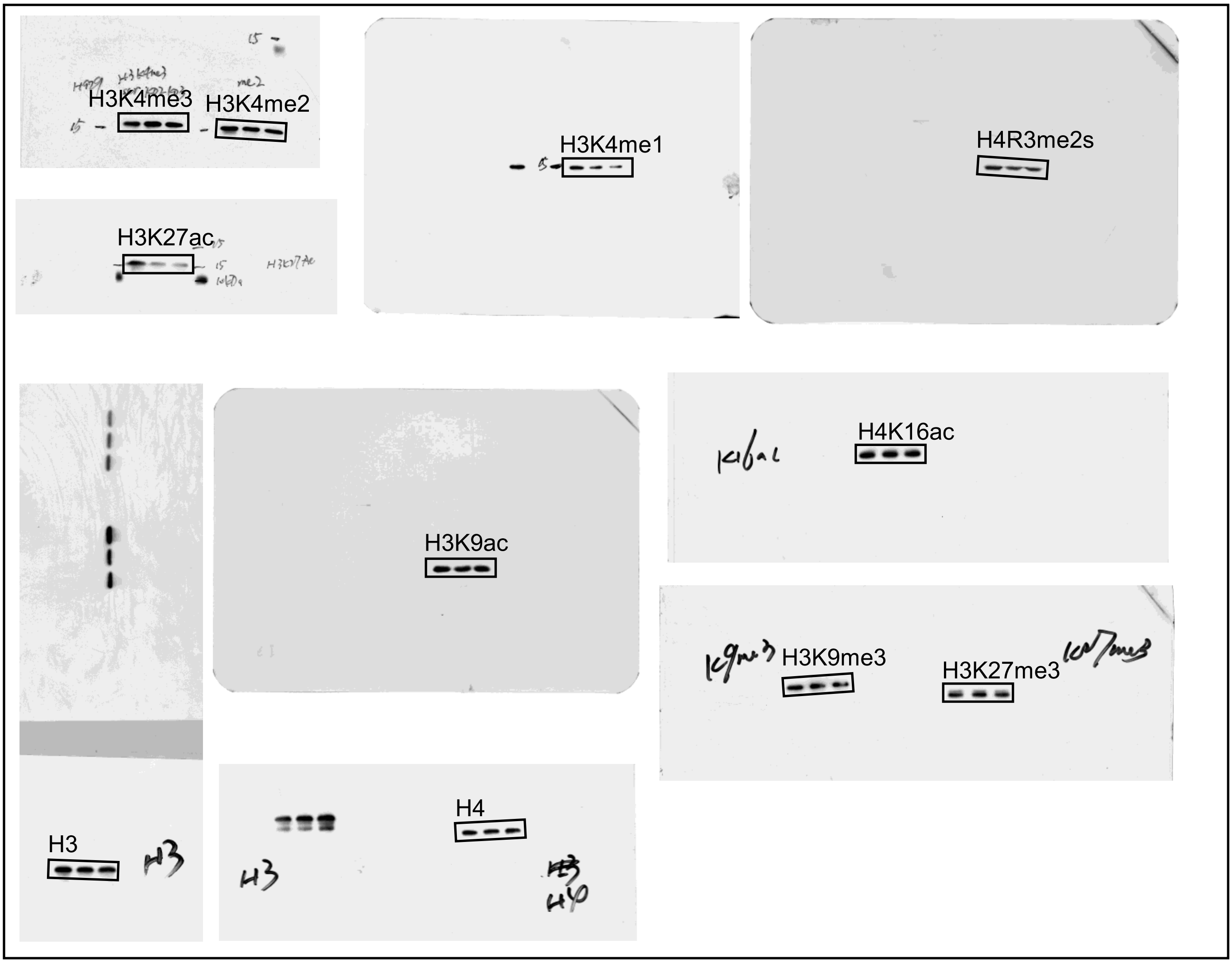

Figure 6A

Tissues

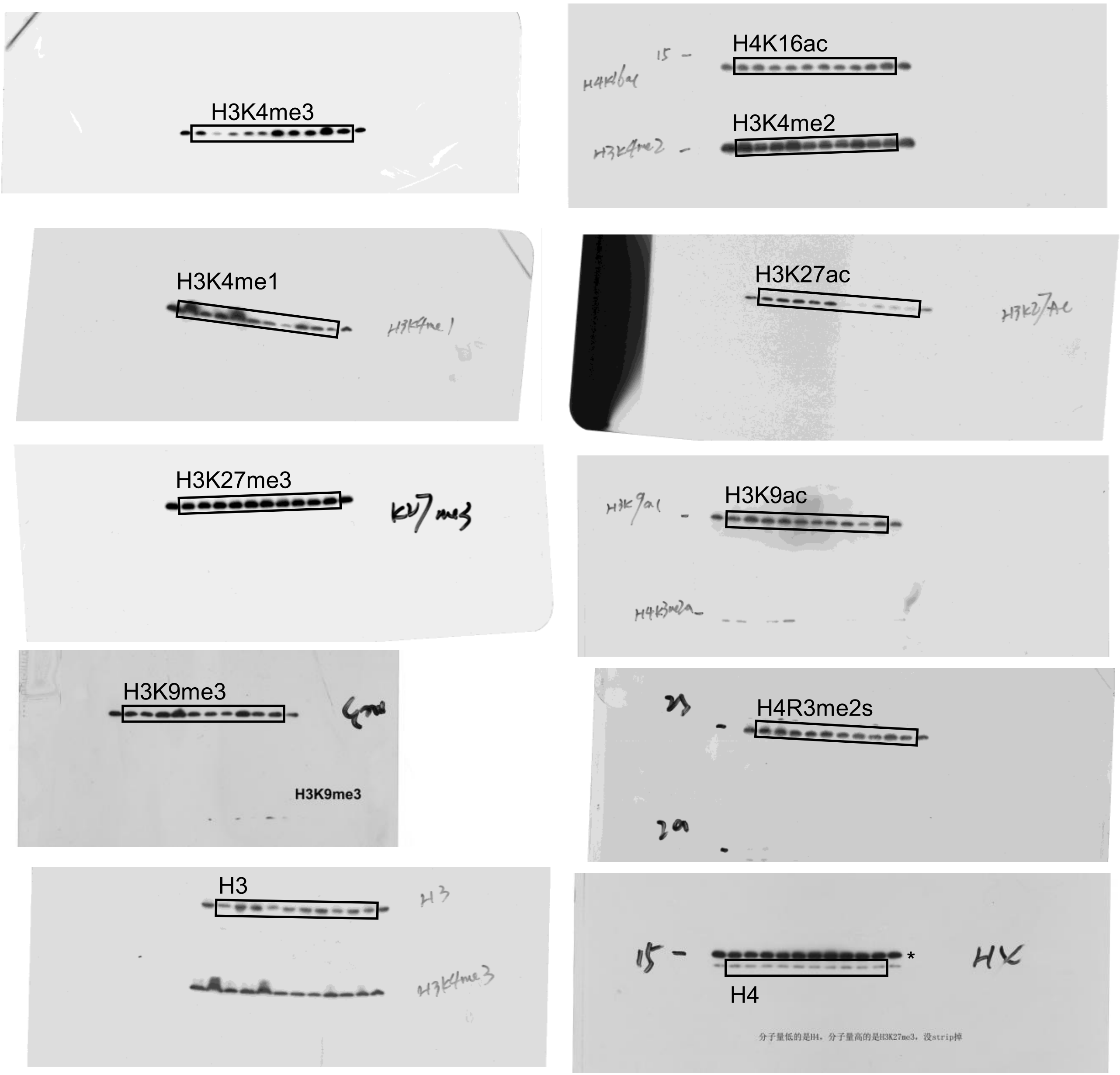

*, The asterisk indicated that the remaining H3K27me3 signal had not been successfully stripped.

Figure 6B

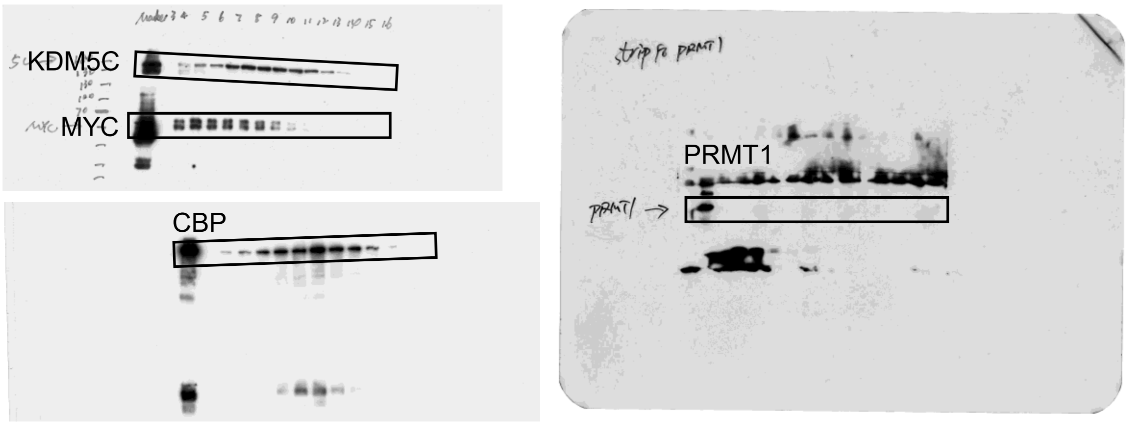

Figure 6C

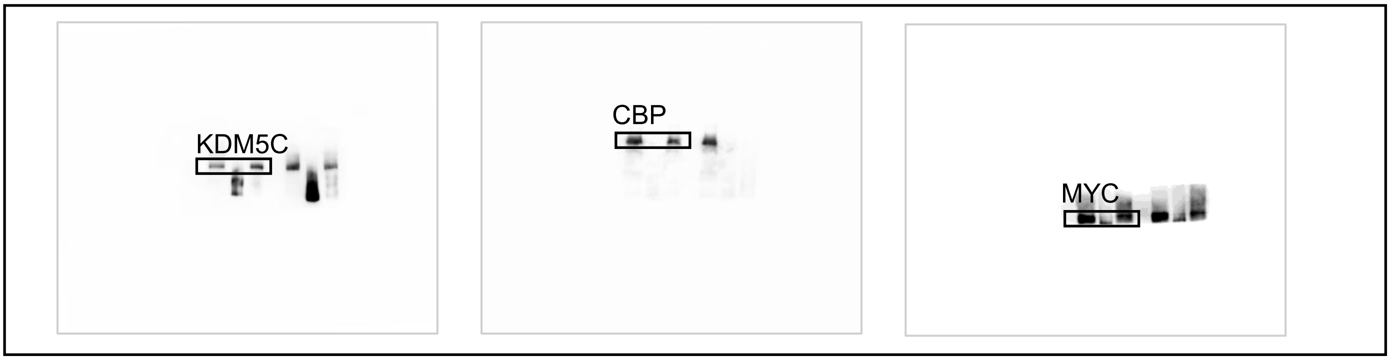

Figure 6D

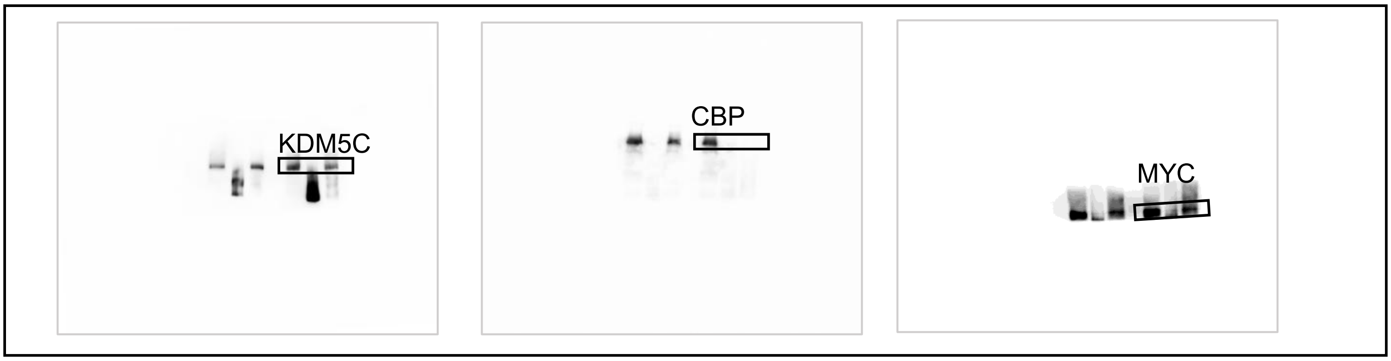

Figure 6F

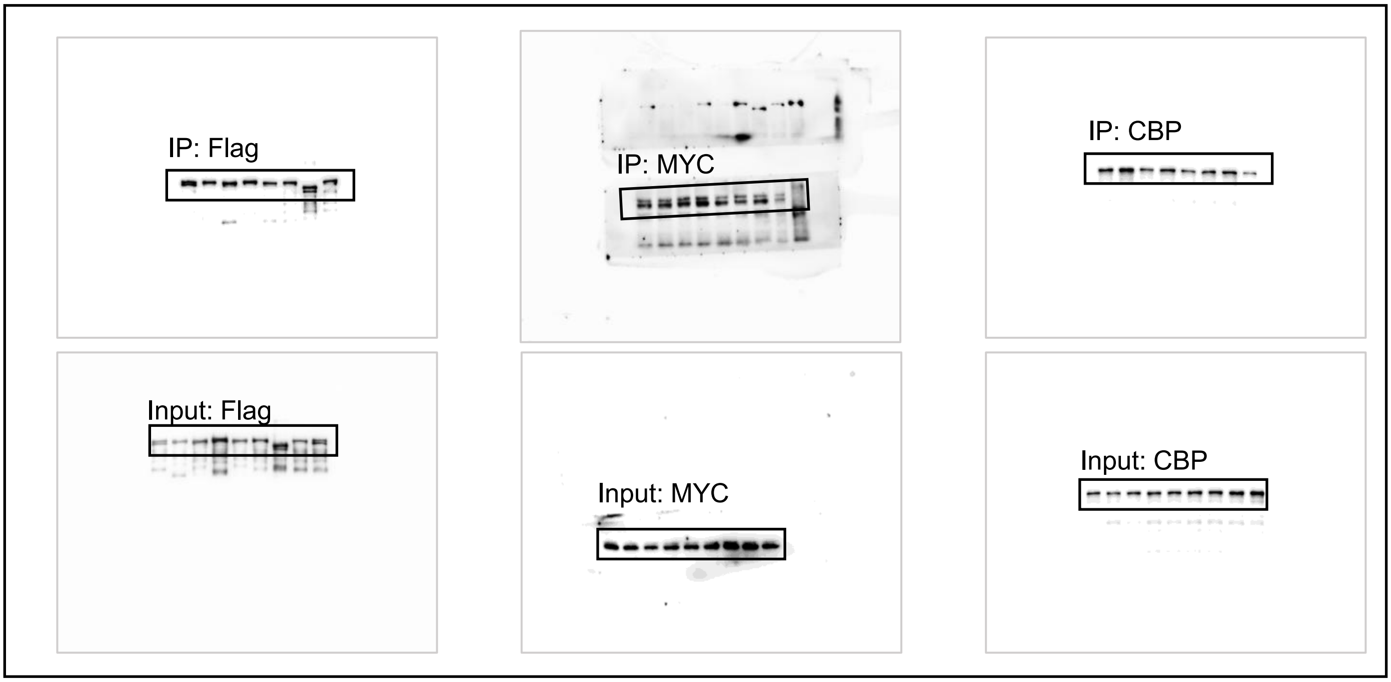

Figure 6H

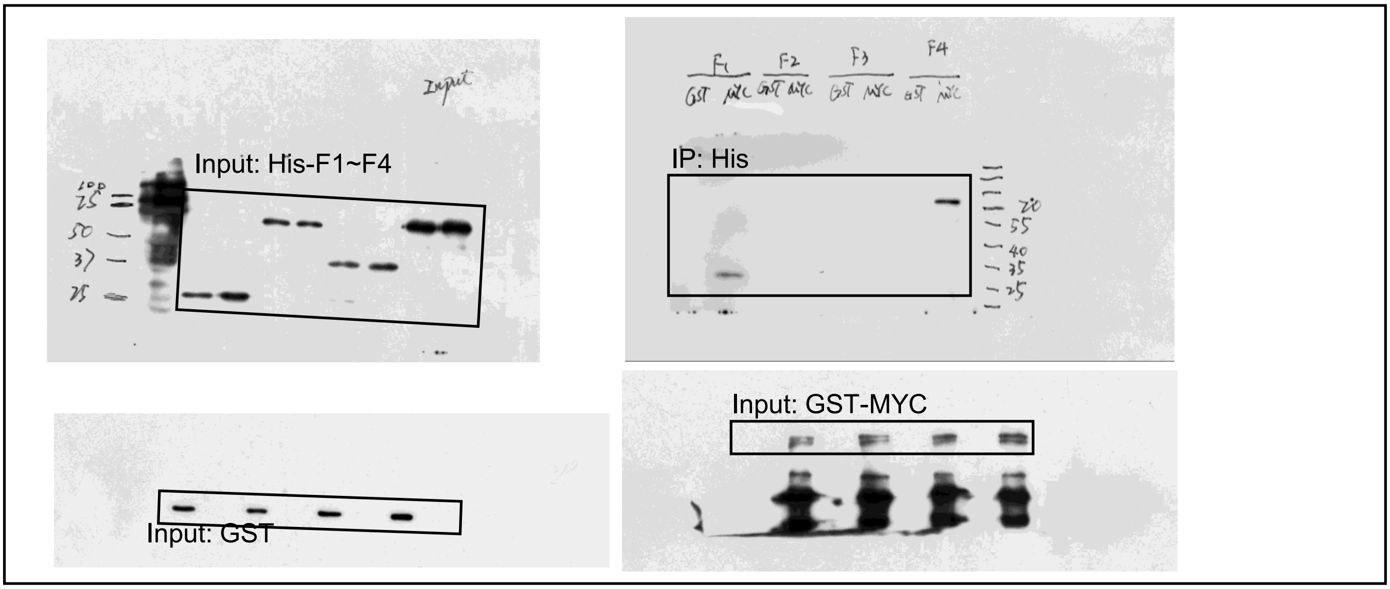

Figure 6J

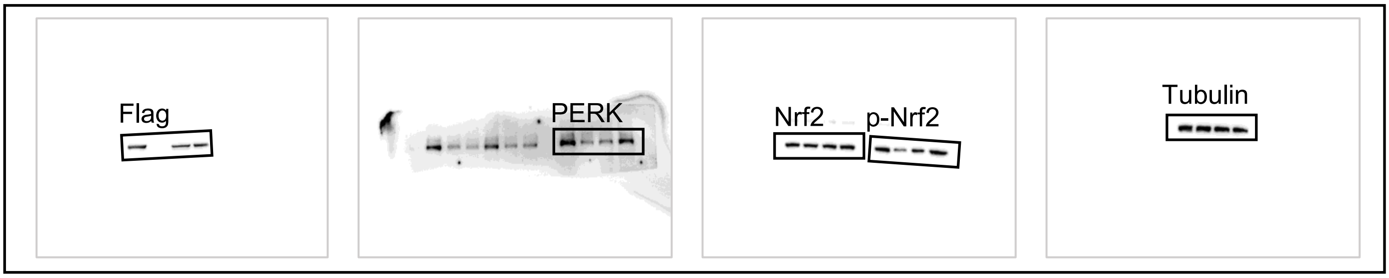

Figure S5A

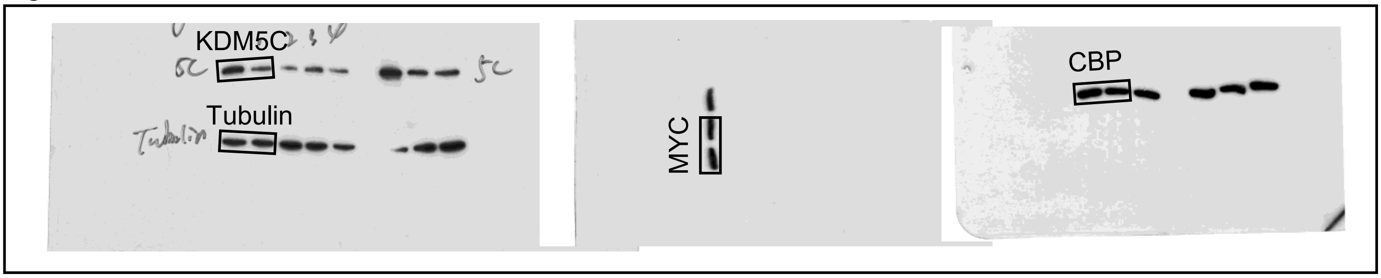

Figure S5B

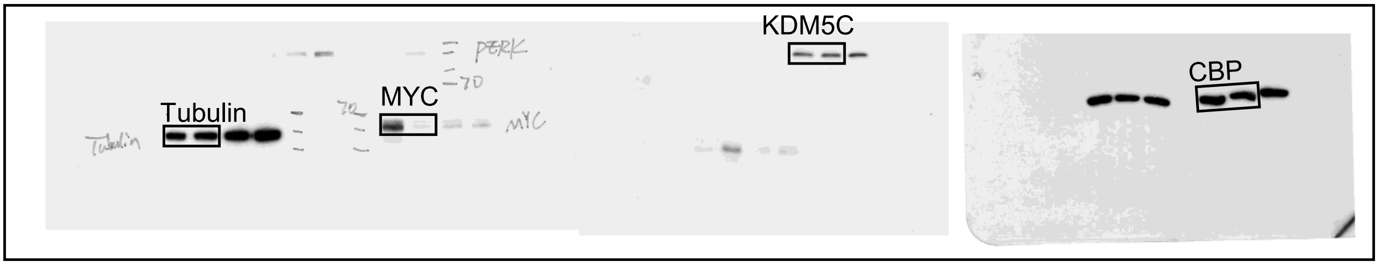

Figure S5C

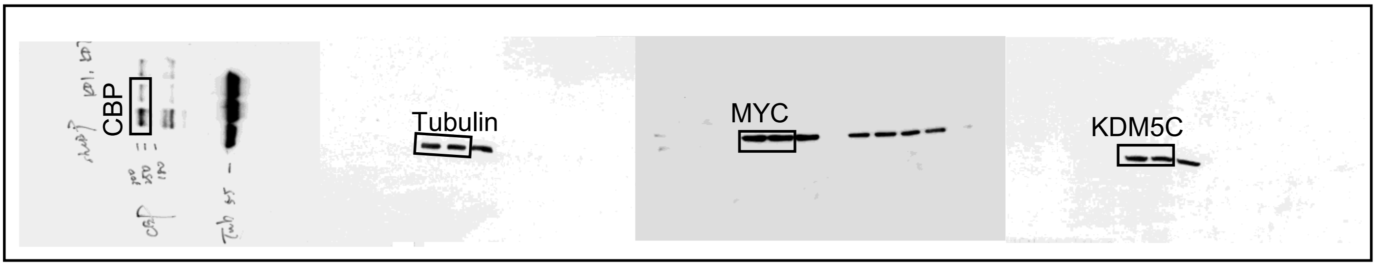

Figure S5E

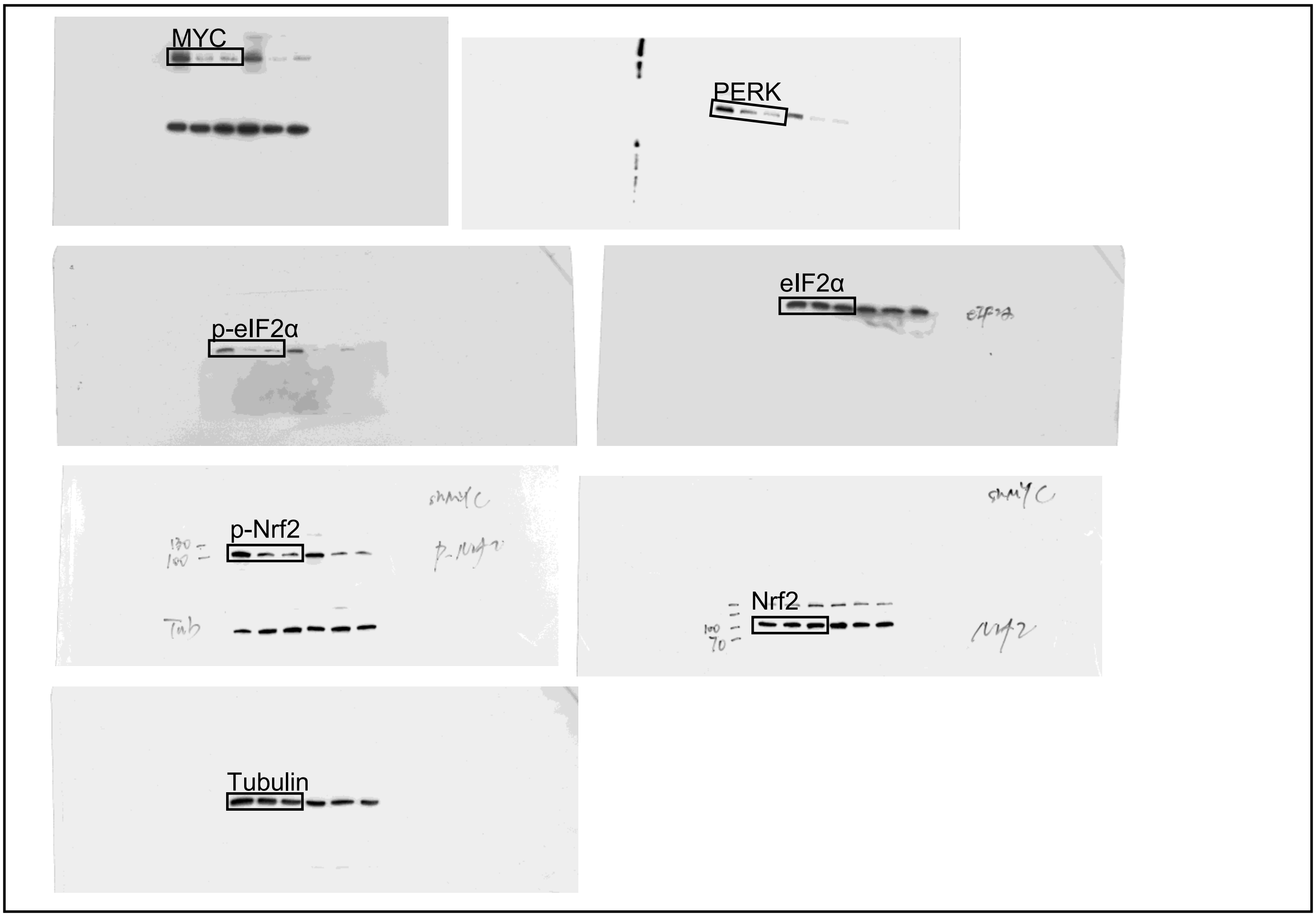

Figure S5G

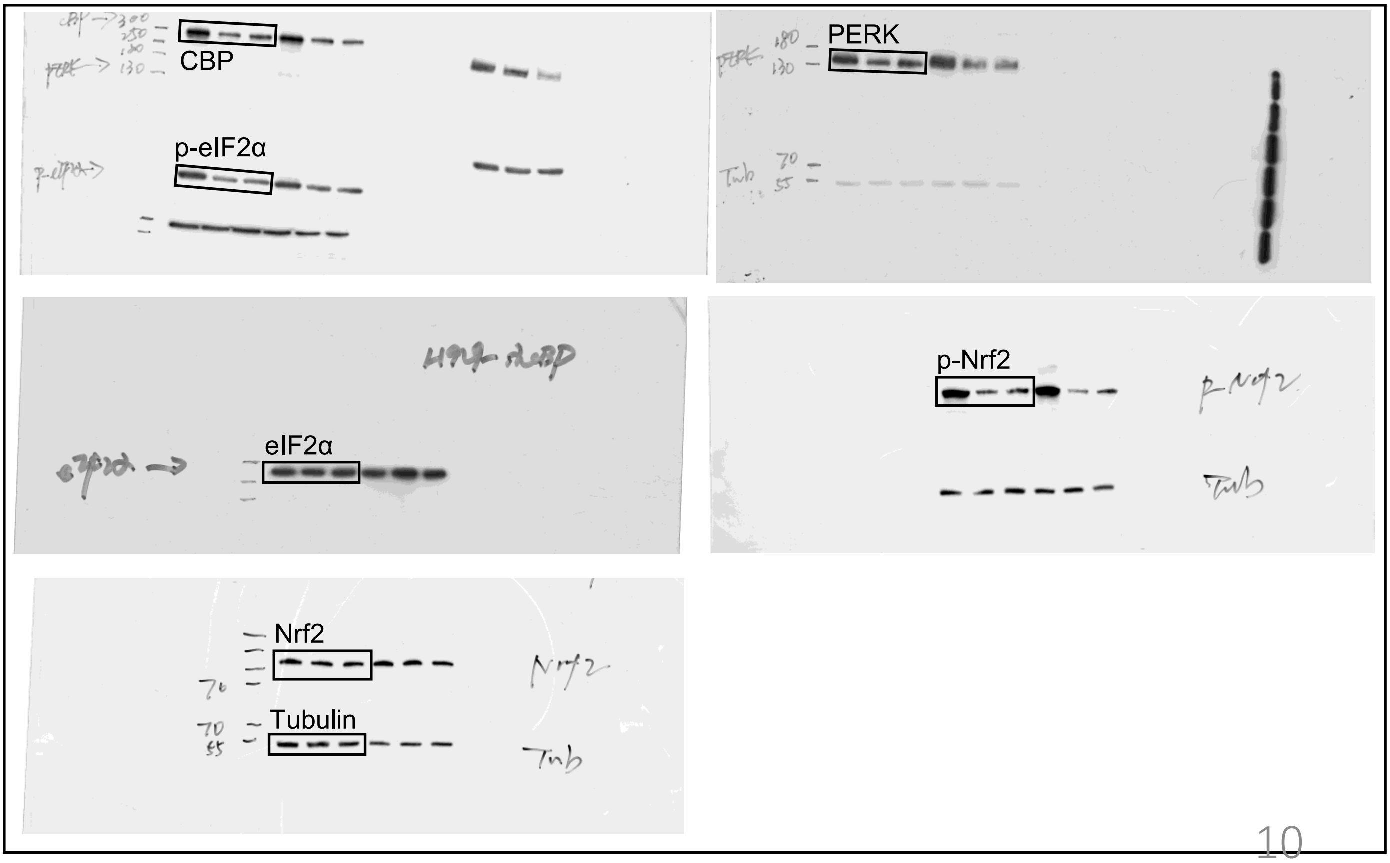

Figure7F

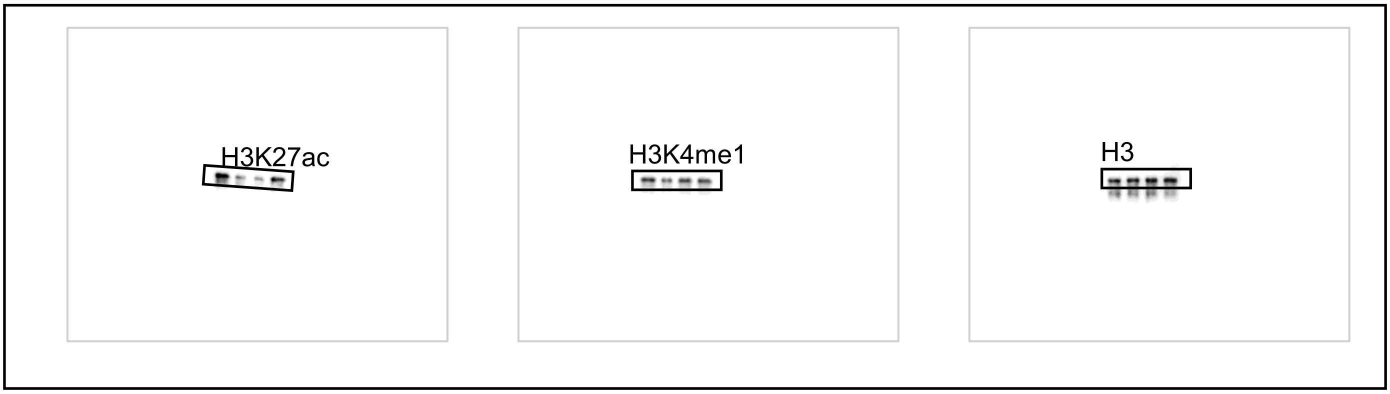

Figure S5I

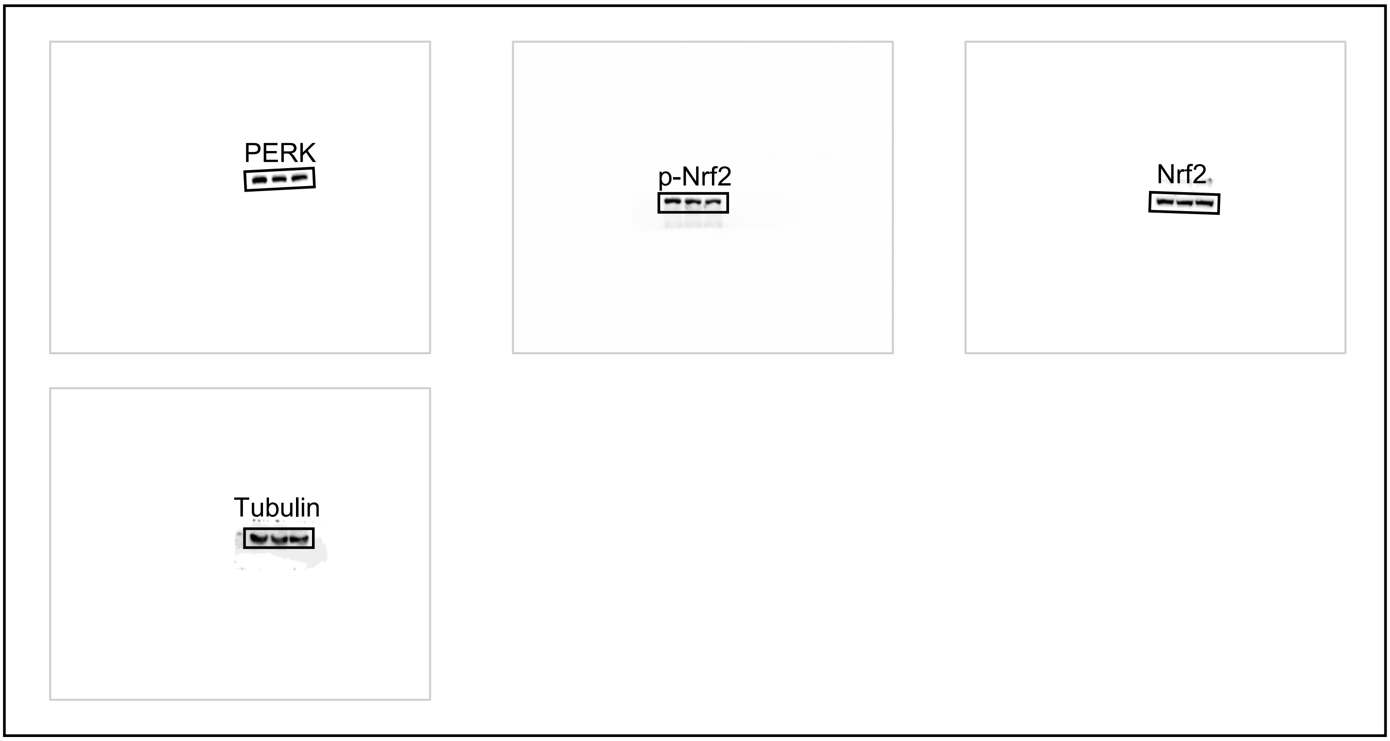

Supplement: Supplementary file 2 — Original experimental data [file 41419_2026_8591_MOESM2_ESM.pdf]
